# Preserving provability over GPU program optimizations with annotation-aware transformations

**DOI:** 10.1007/s10703-025-00480-7

**Published:** 2025-11-22

**Authors:** Ömer Şakar, Mohsen Safari, Marieke Huisman, Anton Wijs

**Affiliations:** 1 Formal Methods and Tools, University of Twente, Enschede, The Netherlands; 2 High-Performance Computing & Visualization, SURF, Amsterdam, The Netherlands; 3 Software Engineering & Technology, Eindhoven University of Technology, Eindhoven, The Netherlands

**Keywords:** GPU, Optimization, Deductive verification, Annotation-aware, Program transformation

## Abstract

GPU programs are widely used in industry. To obtain the best performance, a typical development process involves the manual or semi-automatic application of optimizations prior to compiling the code. Such optimizations can introduce errors. To avoid the introduction of errors, we can augment GPU programs with (pre- and postcondition-style) annotations to capture functional properties. However, keeping these annotations correct when optimizing GPU programs is labor-intensive and error-prone.

This paper presents an approach to automatically apply optimizations to GPU programs while preserving provability by defining *annotation-aware transformations*. It applies frequently-used GPU optimizations, but besides transforming code, it also transforms the annotations. The approach has been implemented in the Alpinist tool and we evaluate Alpinist in combination with the VerCors program verifier, to automatically apply optimizations to a collection of verified programs and reverify them.

## Introduction

Over the course of roughly a decade, graphics processing units (GPUs) have been pushing the computational limits in fields as diverse as computational biology [73], statistics [39], physics [9], astronomy [25], deep learning [32], and formal methods [17, 47, 48, 74, 76]. Dedicated programming languages such as CUDA [37] and OpenCL [46] can be used to write GPU source code. To achieve the most performance out of GPUs, developers apply incremental optimizations, tailored to the GPU architecture. Unfortunately, this is to a large extent a *manual* activity. The fact that for different GPU devices, the same code tends to require a number of transformations [21] makes this procedure even more time consuming and error-prone. Recently, automating this has received some attention, for instance by applying machine learning [4].

**Fig. 1:**
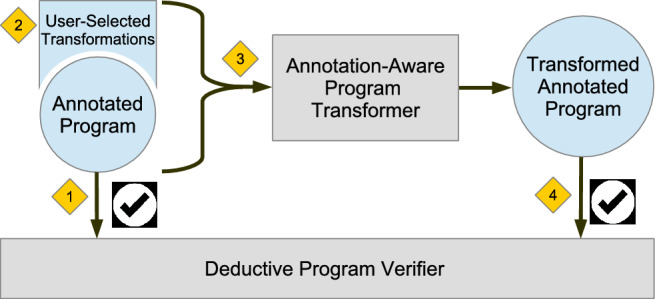
Annotation-Aware Program Transformation.

Reasoning about the correctness of GPU software is hard but necessary, especially when the GPU code is optimized. Multiple verification techniques and tools have been developed to aid in this task aimed at detecting data races, see [10, 11, 15, 35, 36], and for a recent overview, see [22]. Some of these techniques apply deductive program verification, a verification technique based on logical inference, i.e., deduction. This technique requires a program to be *manually* augmented with pre- and postcondition annotations. However, annotating a program is time consuming. The more complex a program is, the more challenging it becomes to annotate it. In particular, as a program is being optimized repeatedly, its annotations tend to change frequently.

This paper presents an approach to automatically apply optimizations to GPU programs while preserving provability by defining *annotation-aware transformations* [29]. These transformations optimize the GPU program while taking the annotations into account and transforming the annotations as well. Interestingly, the presence of annotations can be exploited to determine whether an optimization is actually applicable, and in doing so, it can sometimes apply an optimization where a compiler cannot automatically apply it.

Figure 1 shows a visual representation of the approach. It automates the optimization of GPU code, to the extent that the developer needs to indicate which optimization needs to be applied where, and the tool performs the transformation. As it applies a code transformation, it also transforms the corresponding annotations, which means that once the developer has annotated the unoptimized, simpler code, any further optimized version of that code is automatically annotated with updated pre- and postconditions, making it reverifiable. The result of this annotation-aware transformation is an *optimized program* that can be *reverified* by a deductive program verifier. This avoids having to re-annotate the program every time it is optimized for a specific GPU device.

This approach has been applied to six frequently used optimizations, namely:

1. Loop unrolling: Executing loop iterations before the loop.
2. Iteration merging: Executing multiple loop iterations in one single iteration.
3. Data prefetching: Prefetching data residing in global memory into registers.
4. Matrix linearization: Changing a matrix into an array.
5. Tiling: Increasing the work done by each thread.
6. Kernel fusion: Joining two consecutive kernels.

The approach and the optimizations mentioned above have been implemented in the Alpinist tool. The *functional correctness* of GPU programs [11] and the optimized GPU programs can be verified with the deductive program verifier VerCors. VerCors allows the verification of many typical GPU computations, see e.g., [53, 55, 56]. The current version of Alpinist works with a language called PVL, which is the language of VerCors. However the approach is general and can also be applied to CUDA, OpenCL or other languages.

The contributions of this paper are:

- The approach to automatically apply optimizations to GPU programs while preserving provability by defining *annotation-aware transformations*.
- The application of the approach to the frequently used optimizations mentioned above.
- Alpinist, a tool that can apply the six annotation-aware transformations.

This paper is an extended version of our paper presented at TACAS 2022 [58]. In the TACAS paper, we introduced Alpinist, we discussed loop unrolling, tiling and a part of kernel fusion in detail and mentioned the other optimizations. In this paper, we discuss kernel fusion completely and the other optimizations, namely data prefetching, matrix linearization and iteration merging in detail. We also discuss the evaluation in greater detail.

It should be noted that this work is not focussed on optimizing the source code. The focus of this work is in how the annotations are transformed with those transformations and how to preserve the provability of the output. It is up to the developer to check whether a specific application of an optimization is actually more performant. We do not focus on this part, since that is more the role of an auto tuner. CUDA and OpenCL support in Alpinist are left as future work.

### Outline

Section 2 gives the preliminaries on GPU programs, VerCors and its language PVL. Section 3 demonstrates how the approach optimizes a verified GPU program while preserving its provability using Alpinist. Section 4 discusses the architecture of Alpinist. Section 5 discusses six GPU program optimizations. Section 6 presents the results of experiments in which Alpinist has been applied on a collection of programs. Section 7 discusses related work and Sect. 8 concludes the paper, and discusses future work.

## Preliminary

In this section we introduce the GPU programming model, VerCors and its language PVL.

### GPU programming model

We briefly discuss the different parts of the GPU programming model, namely host and device, threads, memory and synchronization. We leave out details of the GPU architecture that are not relevant for this paper.^1^ In Sect. 2.3, we discuss how these different concepts relate to the different PVL constructs.

The workflow of a GPU program in general is that the *host* (i.e., CPU) invokes a kernel to be executed on the device (i.e., GPU). A *kernel* is a GPU function that is executed for a number of GPU threads specified at the launch of the kernel. The data that the kernel works on is copied from *host memory* (i.e., RAM) into *global (device) memory* (i.e., the GPU’s DRAM).

The threads that execute the kernel have a hierarchical structure, they are structured from top to bottom as follows: grids, thread blocks and threads. *Threads* are the smallest unit of execution in this hierarchy. Each thread has a unique identifier by which the kernel can direct the different threads to different data. These threads are grouped into *thread blocks* and those thread blocks are grouped together into a *grid*.

There are different levels of memory within a GPU, from slowest to fastest memory: global memory, shared memory and registers. Each thread has private, thread-local memory called *registers*. Each thread block has *shared memory* that is shared between all threads within a thread block and can be used for communication between threads. *Global memory* is used for memory exchanges with the host.

There are two ways to synchronize between threads, namely global synchronization and local synchronization. *Global synchronization* is the synchronization over all threads, this is only achieved between kernel invocations. *Local synchronization* is the synchronization of threads within a thread block and is achieved with a (thread-block-local) *barrier*.

### VerCors’ architecture

VerCors is a deductive program verifier, designed to work for different input languages (e.g., Java and OpenCL). It takes as input an annotated program, which is then transformed in several steps into an annotated Viper program, where Viper [41, 69] is an intermediate verification infrastructure. Viper then generates proof obligations, which can be discharged by an automated theorem prover, such as Z3 [40].

The internal transformations in VerCors are defined over our internal *abstract syntax tree* (AST) representation (written in the Common Object Language or COL [57]), which captures the features of all input languages. Some transformations are generic (e.g., splitting composite variable declarations) and others are specific to verification (e.g., transforming contracts). The transformations implemented as part of Alpinist are also applied on the COL AST, but they are developed with a different goal in mind, and in particular several of the transformations are specific to the supported optimizations.

Using VerCors and its architecture to implement Alpinist gives us some benefits. First, existing helper functions can be reused, which simplifies tasks such as gathering information regarding specific AST nodes. Second, some generic transformations of VerCors can be reused, such as splitting composite variable declarations or simplifying expressions. This helps to simplify the implementation of the optimizations. Third, using the architecture of VerCors allows us to prove assertions that we generate relatively easily by invoking VerCors internally.

### PVL

VerCors has its own language PVL. Below we introduce its syntax by example and mention how they relate to GPU program constructs. Note, for readability the examples in this paper are written in a simplified version of PVL.

**Fig. 2:**
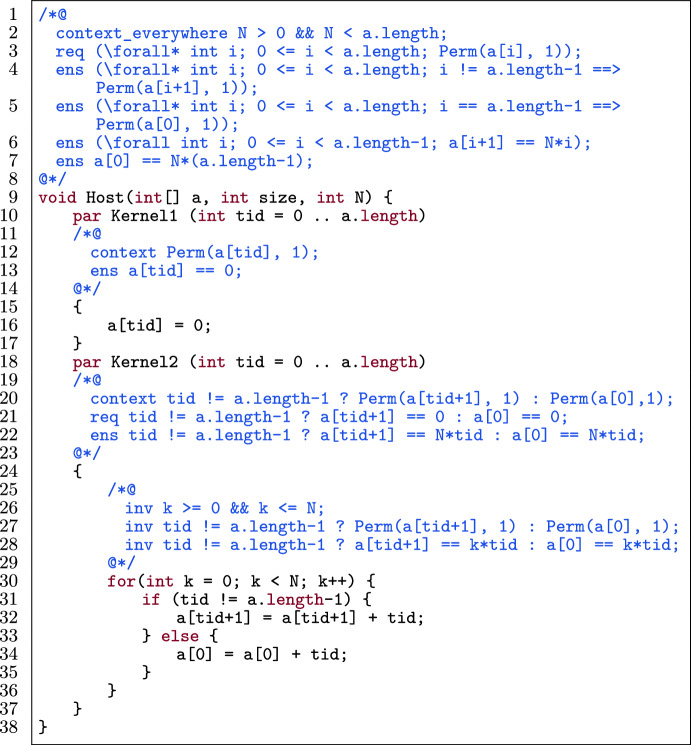
A GPU-style PVL program.

Figure 2 shows a GPU program with annotations. There are two parallel blocks, denoted by the par keyword (l.10, l.18), running a.length threads.^2^ A *parallel block* represents a GPU kernel executed by a single thread block.

The main function Host represents a host function which invokes Kernel1 first, then Kernel2. Variables declared inside of the kernels reside in registers and the variables outside of the kernels reside in global memory.

The kernels, the for-loop and the host function are annotated for verification (in blue), using permission-based separation logic [8, 12, 13]. Permissions capture which memory locations may be accessed by which threads; they are fractional values in the interval (0, 1] (cf. [13]): any fraction in the interval (0, 1) indicates a read permission, while 1 indicates a write permission. A write permission can be split into multiple read permissions and read permissions can be added up, and transformed into a write permission if they add up to 1. The soundness of the logic ensures that a program can only be verified if for each memory location the total number of permissions at any point in time does not exceed 1.

To specify permissions, predicates are used of the form $$\texttt {Perm(L, }\pi \texttt {)}$$ where $$\texttt {L}$$ is a heap location and $$\pi $$ a fractional value in the interval (0, 1] (e.g., 1$$\backslash $$3). Pre- and postconditions, denoted by keywords $$\texttt {req}$$ and $$\texttt {ens}$$, should hold at the beginning and end of an annotated function, respectively. The keyword $$\texttt {context}$$ abbreviates $$\texttt {req}$$ and $$\texttt {ens}$$ (l.12, l.20). The keyword $$\texttt {context}\_\texttt {everywhere}$$ specifies a property that must hold throughout the function (l.2). Note that $$\backslash \texttt {forall*}$$ is used to express a universal separating conjunction over permission predicates (l.3-l.5) [41] and $$\backslash \texttt {forall}$$ is used as standard universal conjunction over logical predicates (l.6). Logical conjunction is denoted by && and separating conjunction in separation logic is denoted by $$**$$.

**Fig. 3:**
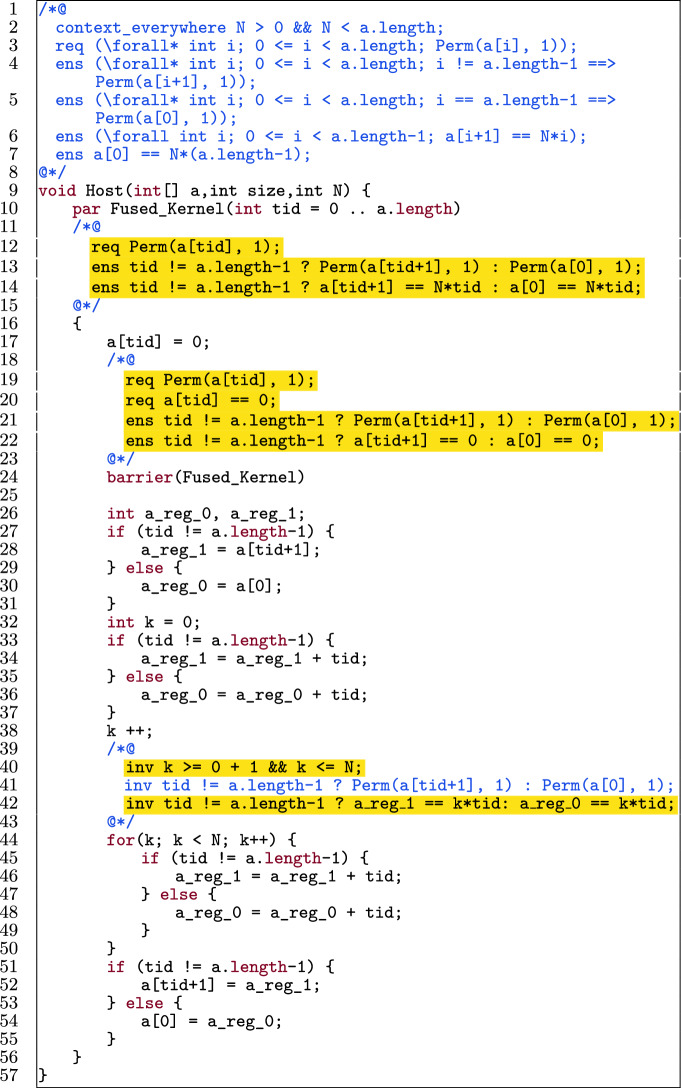
An optimized GPU-style program, annotated for verification.

Figure 3 shows an example of a thread-block-local barrier (l.19–24). The barrier has a contract that specifies the state before and after the barrier. Barriers can be used to redistribute permissions. Note the permissions specified on lines 19 and 21. The barrier requires permission for a[tid] and redistributes this permission over the thread, resulting in either the permission to access a[tid+1] or a[0] depending on the tid.

### The relation between PVL, CUDA, OpenCL & COL

Among VerCors’ supported front ends, PVL has a special relation with COL compared to the other front end languages. COL is meant to be a common language for all front ends. Higher-level concepts, such as kernels in CUDA/OpenCL, are translated into COL, from which point on we have abstracted away from the source program. For kernels in CUDA/OpenCL, these translations to COL abstract away details of the original program while keeping the same semantics. In contrast to PVL, where these translations are an almost one-to-one translation of PVL constructs to COL nodes.

PVL is in a sense defined as the front end for COL. Technically, COL is a language with no concrete syntax, however COL ASTs in this work are presented using simplied PVL, since PVL is the language closest to COL. The optimizations in the following sections are also presented in simplified PVL, since the optimizations are implemented on the COL nodes.

A concrete example is the representation of kernels in COL. COL has a node named ParallelBlock. For PVL, ParallelBlock is represented one-to-one by the par block construct presented above. Using the same ParallelBlock node, an abstract representation of CUDA/OpenCL’s kernels is made, maintaining their semantics.

The current version of Alpinist only supports PVL directly, however since CUDA and OpenCL are translated into COL, the optimizations as presented in this paper are also applicable to CUDA and OpenCL programs, possibly with small changes due to differences in the abstractions.

### GPGPU support in VerCors

The GPU programs that are discussed further in the paper have been verified using VerCors [11]. To understand what VerCors verifies, we summarize the features and properties that VerCors checks. The version of VerCors used by Alpinist is VerCors 1.4 [67].

By default, VerCors checks for memory safety using the permission-related annotations mentioned in the previous section. Two GPU-specific constructs VerCors checks are safety of barriers and atomics.

Thread-local barriers are used for synchronization. This synchronization is needed since the access patterns of threads to heap locations changes after the barrier. Due to the change in access patterns, the permissions given to each thread also needs to change accordingly, specified in the contract of the barrier. If this access pattern does not change, the barrier is redundant. VerCors checks two properties here: 1) whether there is sufficient permission (specified in the precondition) to enter the barrier and 2) whether the postcondition of the barrier only *redistributes* permissions. The second property is of importance, since the barrier cannot ensure more permission (in total) than is specified in the precondition.

Secondly, VerCors has support to reason about atomics [2]. The support is based on the verification approach by He et al. [24], adjusted for GPU programs. Atomics are supported using user-defined invariants. These invariants describe the state of the atomic locations and are checked before and after the atomic operations. Atomics are not used in the optimizations discussed in this paper nor in the examples. Thus, we do not explain them in detail.

VerCors 1.4 does not (fully) support all different aspects of GPGPU programming. Some noteworthy aspects are floating point numbers, multiple thread blocks, warps and shared memory. The floating point numbers are not supported while the other aspects are supported or can be supported with limitations to the verification. These aspects require additional research, we give some insight in the main challenges for all these aspects below.

Regarding the multiple thread blocks, they are represented as nested parallel blocks, however due to limitations in the analysis of non-linear integers for the multi-dimensional thread identifiers and the complexity of the generated universal quantifiers, the support for multiple thread blocks is limited and difficult to use. However, some optimizations discussed in this paper, e.g. kernel fusion, do require knowledge of the thread blocks, e.g. the number and size of the thread blocks. For these cases, the knowledge required by the optimization is established using additional analysis, as is done for the kernel fusion optimization in Sect. 5.6.5. Thus, for the optimizations in this paper we reason about the relevant parts and properties of multiple thread blocks when needed.

Regarding warp support, this should come after proper multiple thread block support. The main challenge in the support for warps is the encoding of the lockstep execution. The GPUVerify tool, a formal verification tool for CUDA and OpenCL kernels has an encoding of warps. Bardsley and Donaldson [6] describe different methods for supporting warps when reasoning about data races. Implementing (one of) these encodings in VerCors is future work.

Regarding shared memory, this should also come after proper multiple thread block support. If multiple thread blocks are encoded as a nested parallel block, then shared memory could be encoded as an array declared in the outer parallel block, making the array specific for each thread block.^3^

Regarding the floating point number support, the main challenge is on the part of the solver rather than engineering work in VerCors. Abstractions can be made such as interpreting floats as real numbers to allow reasoning about floats without overflow and NaN. Proper support for the entire IEEE float specification or subsets of it either requires a solver that can handle the floating point operators or an encoding of the specification, i.e. the underlying bit vectors, the operators and the different modes of floating point operators.

## Annotation-aware transformations: an example

This section illustrates how our approach implemented in Alpinist can optimize a verified GPU program while preserving its provability.

Figure 2 shows a GPU program with annotations [11] that is verified by VerCors. The program initializes an array a, and subsequently updates the values in a, N times. In the first kernel (l.10-l.17), each thread initializes a[tid] to 0. In the second kernel (l.18-l.37), each thread updates a[tid+1] (modulo a.length) N times, by adding tid to it.

In the example, write permissions are required for all locations in a (l.3). The pre- and postconditions of the first kernel specify that each thread needs write permission for a[tid] (l.12). The postcondition states that a[tid] is set to 0 (l.13). In the second kernel, all threads have write permission for a[tid+1], except thread a.length-1 which has write permission for a[0] (l.20). Moreover, it is required that a[tid+1] (modulo a.length) is 0 (l.21). For the for-loop (l.30-l.36), loop invariants are specified: k is in the range $[0,\texttt {N}]$ (l.26), each thread has write permission for a[tid+1] (modulo a.length) (l.27) and this location always has the value k*tid (l.28). The postconditions of the second kernel and the host function are similar to this latter invariant.

Figure 3 shows an optimized version of the program, with updated annotations to make it verifiable. Alpinist has applied three optimizations:

1. *Fusing the two kernels*: as mentioned before, the only *global* synchronization point in GPU programs (used, for instance, to avoid data races) exist implicitly between kernel launches. However, if such a global synchronization point is not really needed between two specific kernels, then fusing them gives several benefits, in particular the ability to store intermediate results in (fast) thread-local register memory as opposed to (slow) GPU global memory, and it has a positive effect on power consumption [71]. In the example, the kernels are combined into Fused_Kernel, and a *thread block-local* barrier is introduced (l.24) to avoid data races within the single thread block executing the code.
2. *Using register memory*; register variables can be used to reduce the number of global memory accesses. Here, the use of a_reg_0 and a_reg_1 has been enabled by kernel fusion (l.26).
3. *Unrolling the for-loop*; the for-loop has been unrolled once here (l.32-l.38). Since GPU threads are very light-weight, compared to CPU threads, any checking of conditions that can be avoided benefits performance. When unrolling a loop, this means that fewer checks of the loop-condition are needed. Note that here, Alpinist benefits from the knowledge that $\texttt {N} > 0$ (l.2), so it knows that the for-loop can be unrolled at least once.

To preserve provability of the optimized program, Alpinist changed the annotations, in particular, it constructed the pre- and postcondition of the fused kernel and the loop invariants (highlighted in Fig. 3). Moreover, Alpinist introduced an annotated barrier (l.18-l.24). Since threads synchronize at a barrier, it is possible to redistribute the permissions. In the rest of the paper, we discuss how our approach tackles these annotation-aware transformations.

## The design of Alpinist

This section gives a high-level overview of the design of Alpinist. The optimizations supported by Alpinist are discussed in Sect. 5.

**Fig. 4:**
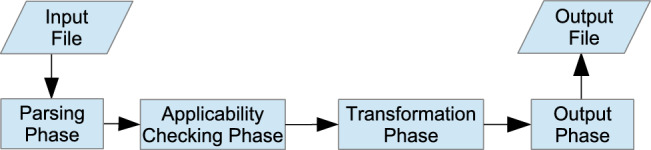
The internal design of Alpinist.

Alpinist  takes a verified file as its input, annotated with special optimization annotations that indicate where specific optimizations should be applied. Currently, the only supported input language is PVL. Alpinist is written in Java and Scala and runs on Windows, Linux and macOS. Figure 4 gives a high-level overview of the internal design of Alpinist. The input program goes through four phases: the *parsing* phase, the *applicability checking* phase, the *transformation* phase and the *output* phase.

The *parsing phase* transforms the input file into a COL AST. Alpinist expects the input program to match a syntactical template for the optimizations to be applied. Section 5 gives these templates for every optimization. If the input program matches the template, Alpinist continues to the applicability checking phase. The *applicability checking* phase performs a check to determine whether the optimization is applicable, that are not captured syntactically by the template. Some optimizations, such as tiling (see Sect. 5.5), are always applicable, hence their applicability check always passes. For other optimizations, prerequisites must be established. Sometimes, a syntactical analysis of the AST suffices, e.g., for kernel fusion (see Sect. 5.6) it must be determined whether there is any data dependency between the two kernels. When analysis of the AST is not enough, VerCors can be used to perform more complex reasoning. An example of this is loop unrolling (see Sect. 5.1). Its prerequisite is that for the loop to be unrollable k times, it is guaranteed that the loop executes at least k times. This prerequisite is encoded as an assertion to be proven by VerCors.

The *applicability checking phase* is one of the strengths of Alpinist. It exploits the fact that the input program is annotated to determine whether an optimization is applicable, and relies on the fact that VerCors can perform complex reasoning. Moreover, this approach allows Alpinist to distinguish between failure due to unsatisfied prerequisites and failure due to mistakes in the transformation procedure.

If the applicability check passes, the transformation phase is next, otherwise a message is generated that the prerequisites could not be proven.

The *transformation* phase applies the optimizations to the input AST. The *output phase* either prints the optimized program in the same language as the input program, which is currently only PVL, or a message is printed, signifying either a failure in optimizing or a verification failure in the applicability checking phase.

### Implementation

Figure 4 reflects the phases an input program goes through in Alpinist. This is also the sequence of phases as presented to the user, e.g. if the applicability checking phase fails, there is no transformed program.

In the implementation, the transformation phase reuses information from the applicability checking phase (where possible) for efficiency. For example, Sect. 5.2 explains an optimization applied on a loop that requires the extent of the loop, i.e. the number of iterations, to be known. The applicability checking phase checks whether the extent of the loop is computable and the transformation phase will compute the extent. Once the applicability checking phase has checked whether the extent is computable, it has all the information needed by the transformation phase. So, instead of executing these phases independently without any communication, these phases are executed at the same time with communication between them.

## GPU optimizations

Alpinist supports six frequently-used GPU optimizations, namely loop unrolling, iteration merging, matrix linearization, data prefetching, tiling and kernel fusion. This section discusses these optimizations in detail. Each optimization is introduced in the context of GPU programs with a general template, detailing the expected structure of the input program. Then, we discuss how to apply them. Interesting insights are discussed where relevant.

### Loop unrolling

Loop unrolling is a frequently-used optimization technique that is applicable to both GPU and CPU programs. It unrolls iterations of a loop, which increases the code size, but can have a positive impact on program performance; e.g., see [21, 26, 42, 50, 66, 72] for its impact, specifically on GPU programs.

**Fig. 5:**
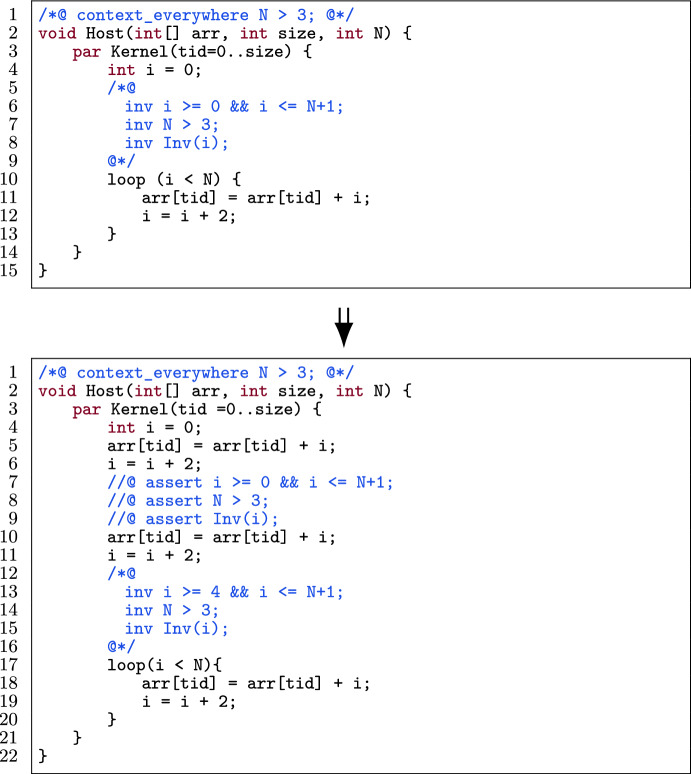
An example of unrolling a loop two times.

Figure 5 shows an example of unrolling an (annotated) loop twice: the body of the loop is duplicated twice before the loop. This has the following effect on the annotations: the loop invariant bounding the loop variable (l.6) changes in the optimized program (l.13).

Note that the other loop invariants (i.e., Inv(i)) remain the same. Moreover, after each unrolling part, we add all invariants as assertions (l.7-l.9) except after the last unroll. These invariants capture the fact that the code produced by unrolling the loop should still satisfy the original loop invariants.

Our approach to loop unrolling is more general than optimization techniques during compilation. For instance, the unroll pragma in CUDA [62] and the unroll function in Halide [63] unroll loops by calculating the number of iterations to see if unrolling is possible, i.e., it should be computable at compile time. This difference is illustrated in Fig. 5 where N (i.e., the number of iterations) is unknown at compile time. Both Halide’s and CUDA’s approach *cannot automatically* handle this case, while our approach *can automatically* unroll the loop, since annotations (l.1, l.8) specify the lower bound of N (provided by the programmer, who knows that this is a valid lower bound). VerCors verifies that the unrolling is valid.

#### Template

**Fig. 6:**
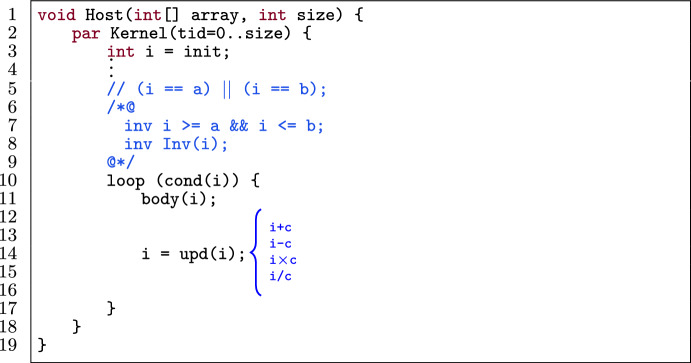
A kernel template for loop unrolling.

Figure 6 shows a template of a loop in a verified GPU program. The loop (l.10) has an induction variable i (l.3) and a sequence of statements in the i^th^ iteration body(i) (l.11). The variable i has a lower bound a and upper bound b which is expressed as a loop invariant (l.7). The initial value of i is either its lower bound or upper bound (l.5). The update statement on i (l.14) is restricted to the expressions $(\texttt {i} + \texttt {c})$, $(\texttt {i} - \texttt {c})$, $(\texttt {i} \times \texttt {c})$ or $(\texttt {i} / \texttt {c})$ where c is a positive integer constant.^4^

#### Transformation

**Fig. 7:**
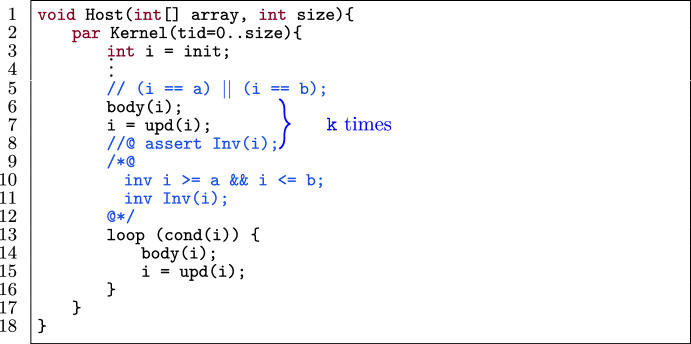
Application of loop unrolling on the template in Fig. 6.

Figure 7 shows the application of loop unrolling (with k unrolls) on the template in Fig. 6 while preserving the provability of the program. To accomplish this, we follow a procedure consisting of three stages: the main, checking and updating stage.

The *main stage* takes as the input of this optimization an annotated (verified) GPU program matching the template in Fig. 6 and a positive unrolling factor k. Next, the *checking stage* checks whether it is possible to unroll the loop k times.^5^

This part corresponds with the applicability checking phase. Thus, we statically calculate the number of loop iterations, by counting how many times the condition (cond(i)) holds starting from either a (as the lower bound of i) or b (as the upper bound of i), depending on the operation of upd(i). If k is greater than the total number of loop iterations at the end of the checking part, then we report an error. Otherwise we go to the *updating part*, in which we update the initial value of i according to the operation in upd(i). If the operation is addition or multiplication, then the loop variable i (in the unoptimized program) goes from a to b. That means, after unrolling, a should be updated according to the constant c from the update expression and k. If the operation is subtraction or division, i goes from b to a. Thus, after unrolling, b should be updated.

**Fig. 8:**
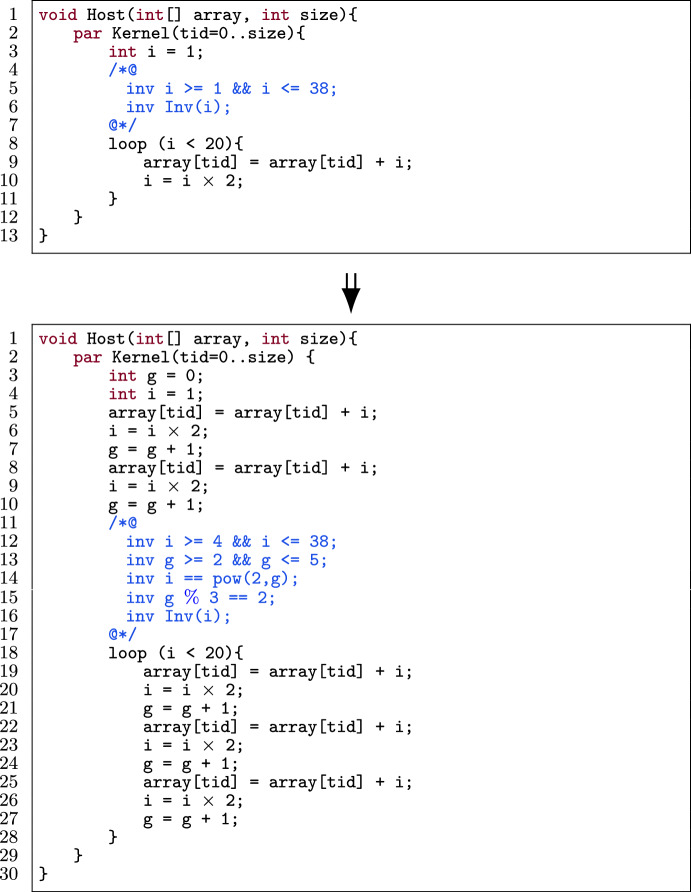
An example of merging 3 iterations.

After the updating part, we return to the main part to unroll the loop k times (l.6–8).

### Iteration merging

Iteration merging is another optimization technique that is applicable to both GPU and CPU programs.^6^ In this optimization, we merge some iterations of the loop into one iteration. Therefore, there are fewer iterations in the optimized program, which leads to fewer comparison operations in the loop. Iteration merging can have a positive performance impact; see [26, 42, 50, 60] for the effectiveness of this optimization on GPU programs.

**Fig. 9:**
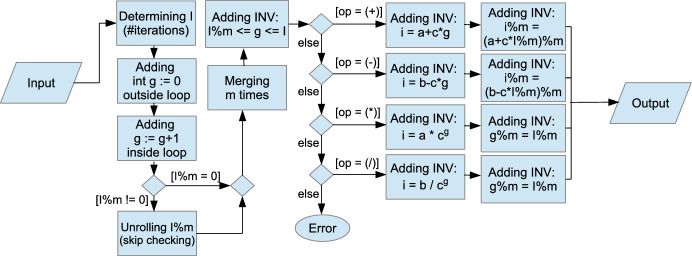
General procedure of iteration merging optimization.

In this optimization, an unoptimized verified GPU program (e.g., Fig. 8) and m as the number of loop iterations to be merged are given as input. Figure 8 shows a concrete example of merging a loop three times^7^ and Fig. 9 illustrates the automated procedure of iteration merging while preserving the provability.

**Fig. 10:**
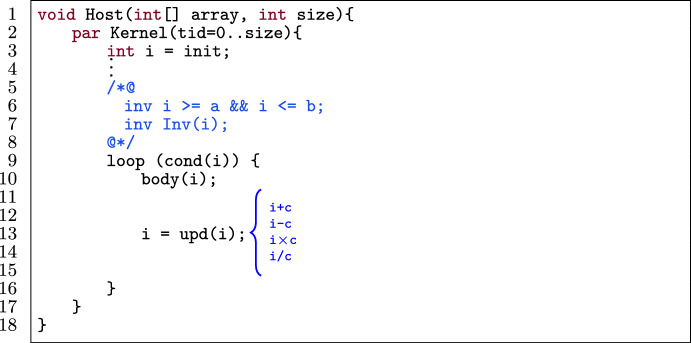
A kernel template for iteration merging.

#### Template

Figure 10 shows a template of a loop in a verified GPU program. The loop (l.9) has an induction variable i (l.3) and a sequence of statements in the i^th^ iteration body(i) (l.10). The variable i is has a lower bound a and upper bound b which is expressed as a loop invariant (l.6). The initial value of i is either its lower bound or upper bound (l.3). The update statement on i (l.13) is restricted to the expressions $(\texttt {i} + \texttt {c})$, $(\texttt {i} - \texttt {c})$, $(\texttt {i} \times \texttt {c})$ or $(\texttt {i} / \texttt {c})$ where c is a positive integer constant.

### Transformation

**Fig. 11:**
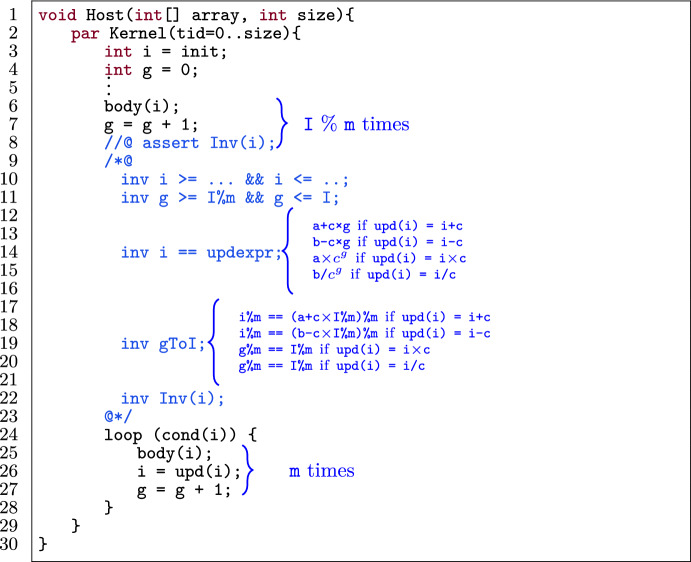
Application of iteration merging on the template in Fig. 10.

Figure 11 shows the application of iteration merging (with m mergings) on the template in Fig. 10 while preserving the provability of the program. Figure 9 illustrates the general procedure for all operators in upd(i).

First we statically determine the (constant) number of loop iterations (I). If I is not a multiple of m, we first have to unroll the loop I % m times (l.6–8), identical to loop unrolling without the checking phase since the number of iterations was already calculated in the first step. Then we merge m iterations of the loop into one. That means we duplicate the body of the loop m times in a row (l.25–27).

To reverify the optimized program, we have to consider the following, subtle problem. Consider the kernel in Fig. 8 to illustrate the problem. In the original program (Fig. 8 (top)) the upper bound for i is specified to be 38, because for static program verification, the largest possible value for i where the loop will execute is 19 (thus 38 after the loop body). However, during the program execution, i will never be more than 16 when the loop body is executed, and thus the actual upper bound on i is 32. The situation is more complex in the optimized program. In Fig. 8, we merge three iterations, hence three update statements (i.e., i = i $\times$ 2). That means we cannot establish the invariant for the upper bound in the optimized program (line 11), as 19 (statically worst case) updated thrice is $19 \times 2 \times 2 \times 2 = 152$ which is greater than 38.

To tackle this problem, we add a ghost variable^8^ to keep track of the number of loop iterations, and generate loop invariants that capture the real number of iterations more precisely. Figure 11 shows this concretely, where Alpinist declares a ghost variable g outside the loop which is initially set to zero (l.4). Then this ghost variable is incremented inside the loop (l.27). We also add three invariants (l.11–19) that relate g to i. The first invariant (l.11) constrains g to start at zero and have an upper bound of I (i.e., the number of iterations). The second and third invariants (l.14 and 19) express how the value of i relates to g, which is dependent on the update expression upd(i):

- If upd(i) = i+c,  we add the invariants  $\mathtt {i == a+c\times g}$  and  $\mathtt {i\%m ==}$ $\mathtt {(a+c \times I\%m)\%m}$
- If upd(i) = i-c,  we add the invariants  $$\mathtt {i == b-c \times g}$$  and  $$\mathtt {i\%m ==}$$$$\mathtt {(b-c \times I\%m)\%m}$$
- If upd(i) = $\texttt {i}\times \texttt {c}$, we add the invariants i == a$\times \texttt {c}^{\texttt {g}}$ and g%m == I%m
- If upd(i) = $\texttt {i}/\texttt {c}$, we add the invariants i == b/$\texttt {c}^{\texttt {g}}$ and g%m == I%m

The first invariant mentioned above expresses the value of i after each iteration in the original loop. For example, the expression $\mathtt {i == a+c \times g}$ can be read as "the initial value of i (i.e., a) plus how many times the constant c has been added to it (i.e., g times)". The second invariant puts an additional constraint to express the value of i (and g) after each iteration in the optimized loop. For example, g%m == I%m can be read as "g should always have the same remainder as I modulo m". We derive this invariant from the fact that initially g starts at I%m since the body has been unrolled I%m times. In every consecutive iteration of the optimized loop, g is increased by m, since we merge m iterations. Thus, initially g%m == I%m holds and after one iteration (g+m)%m == g%m == I%m holds, meaning that it should stay constant. By adding these invariants, we establish the invariant for the lower/upper bound of i as in the original program.

### Data prefetching

**Fig. 12:**
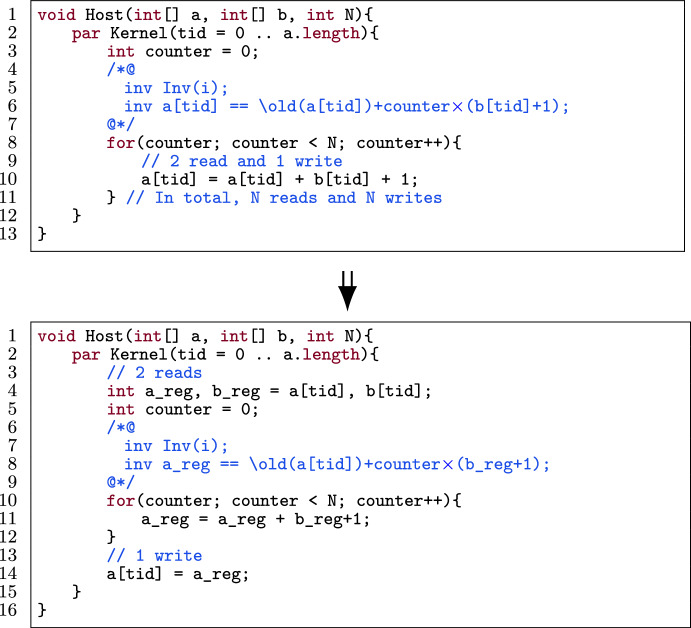
An example of prefetching a[tid] and b[tid].

Suppose there is a verified GPU program where each thread accesses an array location in global memory multiple times. In this optimization, we prefetch the values of those locations that are in global memory into registers which are local to each thread. Therefore instead of multiple accesses to the high latency global memory, we benefit from low-latency registers.

Figure 12 shows an example of data prefetching for two array locations a[tid] and b[tid]. The locations are put into registers (l.4 (bottom)), accesses to the locations are replaced by their register counterparts and written back to the locations (l.14). Data prefetching can have a positive performance impact; see [5, 26, 65, 79].

#### Template

**Fig. 13:**
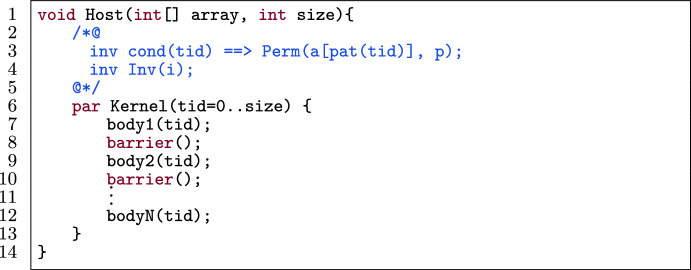
A kernel template for data prefetching.

Figure 13 shows a template of a kernel in a verified GPU program. The kernel has N bodies body1(tid) to bodyN(tid) (l.7–12) separated by barriers (l.8 and 10). The bodies are a sequence of instructions that possibly read/write from/to an array a in global memory with some access pattern pat(tid). Examples of access patterns are a[tid], a[tid+1] and a[0]. If N is 1 (i.e., there are no barriers), then the barrier and subsequent bodies in the template can be ignored. The permission for a[pat(tid)] (l.3) is guarded by a condition cond(tid) that expresses which threads have permission to that pattern. If there is no such condition, then cond(tid) is considered true.

#### Transformation

**Fig. 14:**
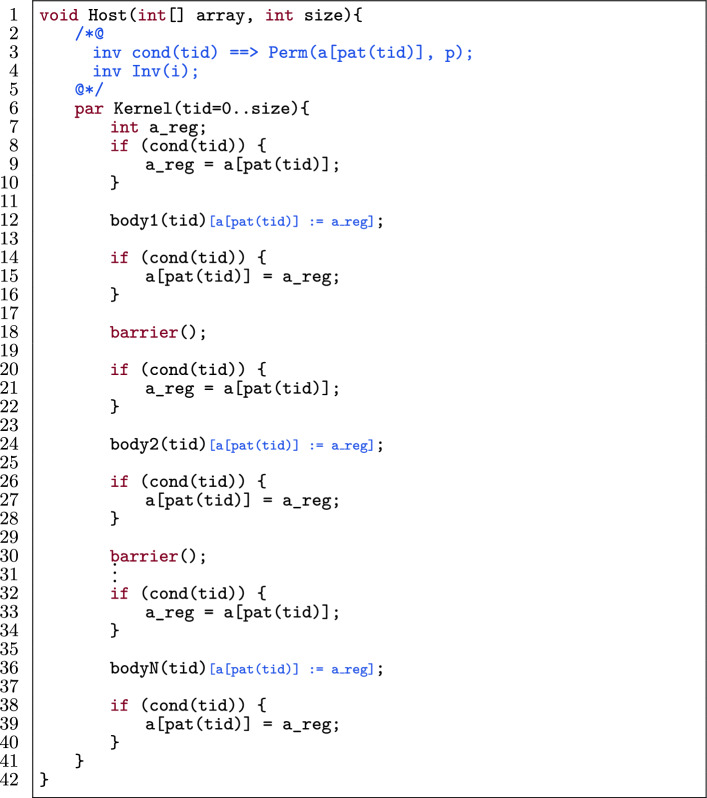
Application of data prefetching (on a[pat(tid)]) on the template in Fig. 13.

Figure 14 shows Fig. 13 with data prefetching applied for a[pat(tid)]. Each body of the original template (body1(tid) to bodyN(tid)) is transformed separately. The procedure for each body is as follows:

- The array location a[pat(tid)] is assigned to a register a_reg (l.8–10), under the condition cond(tid) as expressed in the permission for that location (l.3).
- Each access to a[pat(tid)] in bodyi(tid) is:
  - Replaced by a_reg in the code.
  - Replaced by a_reg in the annotations if it is not in an $\backslash$old expression or a permission predicate.
- If a[pat(tid)] is written to in the original body, then the final value of a_reg is assigned to a[pat(tid)] (l.14–16).

**Fig. 15:**
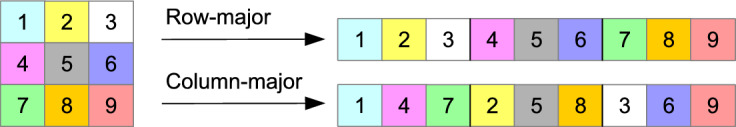
Row-major and column-major representation of a matrix.

**Fig. 16:**
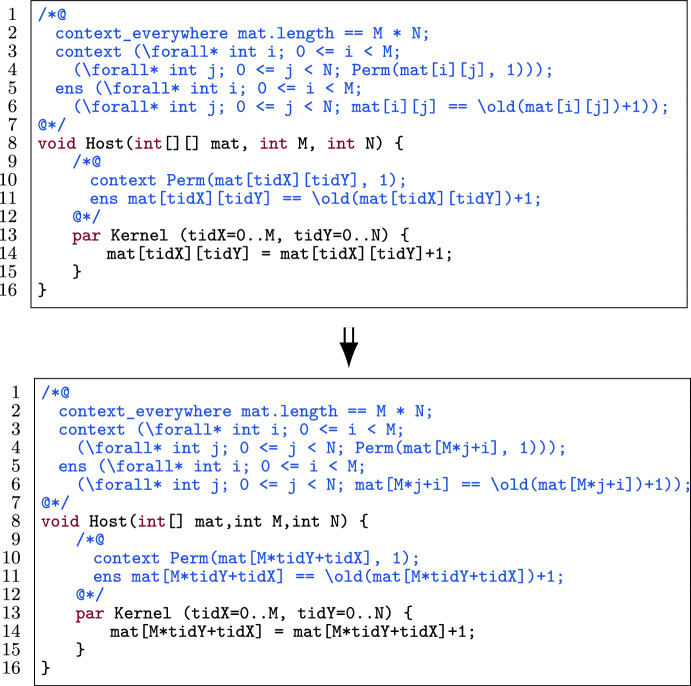
An example of linearizing the matrix mat with column-major access and dimensions M and N.

The $\backslash$old expression and permission predicates are not transformed because (1) the $\backslash$old expressions refer to the values of the locations before the kernel/function, which is not defined for thread-private registers; (2) the permission predicates can only be used for heap locations and not for registers.

### Matrix linearization

Matrix linearization is an optimization where we transform two-dimensional arrays into one-dimensional arrays. Since the physical memory address is linear (i.e., one-dimensional), this optimization might improve memory access patterns; e.g., see [7, 14, 26, 61] for the impact of matrix linearization on GPU programs.

There are two standard techniques to linearize a matrix: 1) a row-major representation where we transform a matrix into an array by storing all elements row by row and 2) a column-major representation where the elements are stored column by column (Fig. 15).

Figure 16 shows an example of how to automatically linearize a matrix with column-major access while preserving the provability of the GPU program. In this figure, we see that both the code and the annotations need to be transformed (such as l.11 for the annotations and l.14 for the code). Note that the number of threads is the same in the original and the optimized program.

#### Template

**Fig. 17:**
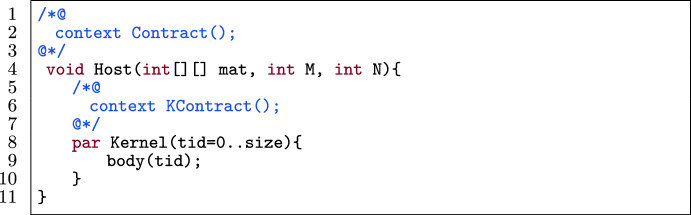
A kernel template for matrix linearization.

**Fig. 18:**
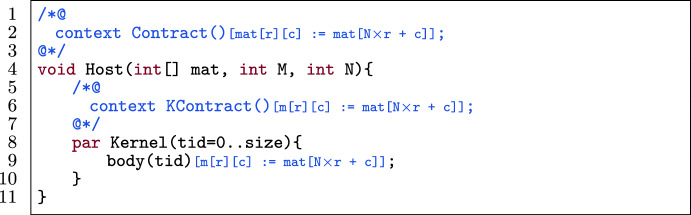
Application of row-major matrix linearization on the template in Fig. 17.

Figure 17 shows a template of a kernel in a verified GPU program. The host function (l.4) has a contract Contract() (l.2) and has as its arguments an integer matrix mat, the number of rows M and the number of columns N. The kernel (l.8) has a body body(tid) (l.9) and contract KContract(tid) (l.6). Contract(), KContract(tid) and body(tid) contain expressions that (partially) match mat[r][c] which accesses the $\texttt {r}^{th}$ row and $\texttt {c}^{th}$ column of the matrix.

#### Transformation

**Fig. 19:**
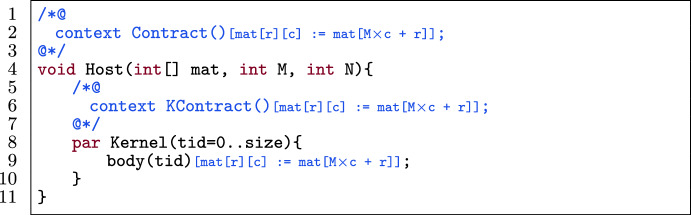
Application of column-major matrix linearization on the template in Fig. 17.

Figures 18 and 19 linearize the matrix in Fig. 17 with row-major and column-major access respectively. The procedure to automatically linearize a matrix mat (M rows, N columns) in a kernel with two-dimensional thread blocks (where each thread has a pair of identifiers r and c) is as follows:

- Change the declaration of mat to a declaration of a one-dimensional array (l.4) with the same name.
- Substitute all accesses of mat[r][c] to mat[N $\times$ r + c] (for row-major access) or mat[M $\times$ c + r] (for column-major access) in both code and annotations, i.e., in Contract() (l.2), KContract(tid) (l.6) and body(tid) (l.9).

### Tiling

**Fig. 20:**
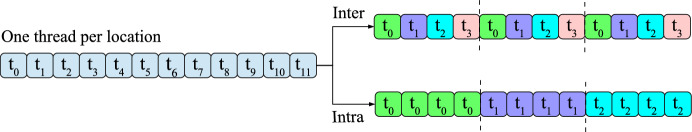
Inter- and intra-tiling of an array as T = 12, N = 4 and $\lceil$T/N$\rceil$ = 3.

**Fig. 21:**
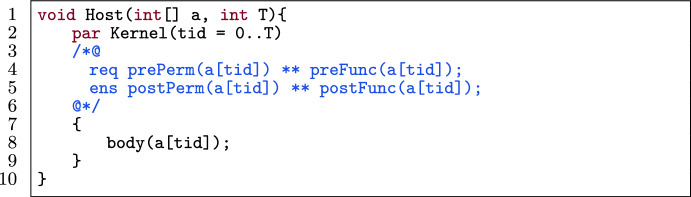
A general unoptimized GPU program to apply for tiling.

Tiling is another well-known optimization technique for GPU programs. It increases the workload of the threads to fully utilize GPU resources by assigning more data to each thread. Concretely, we assume there are T threads and a one-dimensional array of size T in the unoptimized GPU program where each thread is responsible for one location in that array (Fig. 21). To apply the optimization, we first divide the array into $\lceil$T/N$\rceil$ chunks, each of size N (1 $\le$ N $\le$ T)^9^. There are two different ways to create and assign threads to array cells (as in Fig. 20):

- *Inter-Tiling* We define N threads and assign them to one specific location in each chunk. That means each thread serially iterates over all chunks to be responsible for a specific location in each chunk.
- *Intra-Tiling* We define $\lceil$T/N$\rceil$ threads and assign one thread to one chunk (i.e., 1-to-1 mapping) to serially iterate over all cells in that chunk.

Both forms of tiling can have a positive impact on GPU program performance; e.g., see [26, 27, 30, 51, 78] for the impact of these optimizations.

#### Template

Figure 21 shows a template of a kernel in a verified GPU program. The kernel (l.2) has a body body(tid) (l.8). The preconditions of the kernel’s contract are split into permission-related preconditions prePerm(_), and functional-correctness related preconditions preFunc(_) (l.4). The same holds for postconditions (l.5).

#### Transformation

**Fig. 22:**
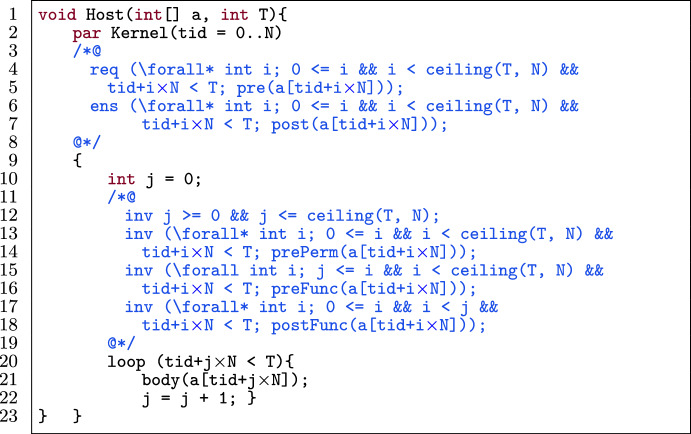
Application of inter-tiling on the template in Fig. 21.

Figure 22 shows the optimized version of Fig. 21 by applying inter-tiling. Regarding program optimization, two major changes happen: 1) the total number of threads has reduced (l.2), and 2) the body is encapsulated inside a loop (l.20-l.22). As mentioned, in inter-tiling, we define N threads instead of T. The number of chunks is indicated by the function ceiling(T, N). Each thread in the newly added loop iterates over all chunks (in the range 0 to ceiling(T, N)-1) to be responsible for a specific location. This happens by the loop variable j and the loop condition $\texttt {tid+j}\times \texttt {N < T}$. This means, each thread tid can access its own location at index tid in each chunk. To preserve verifiability, we add invariants to the loop (l.12-l.19). Therefore, we specify:

- The boundaries of the loop variable j, which iterates over all chunks (l.12).
- A permission-related invariant for each thread in each chunk (l.13). This comes from the kernel’s precondition and is quantified over all chunks.
- An invariant to indicate functional properties of the locations that have not yet been updated by threads in the body of the loop (l.15). This comes from the functional precondition of the kernel and is quantified over all chunks.
- An invariant to specify how each thread updates the array in each chunk (l.17). This comes from the functional property as the postcondition of the kernel and is quantified over all chunks.

Moreover, we modify the specification of the kernel (l.4-l.6). Note that we have the condition $\texttt {tid+j}\times \texttt {N < T}$ in all universally quantified invariants, because the last chunk might have fewer cells than N. We quantified the pre- and postcondition of the kernel over the chunks in the same way as the invariants.

**Fig. 23:**
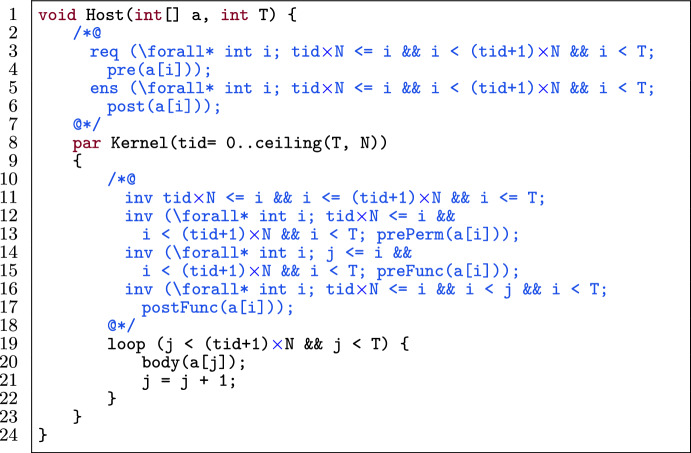
Application of intra-tiling on the template in Fig. 21.

Figure 23 shows the optimized version of Fig. 21 by applying intra-tiling. Intra-tiling is similar to inter-tiling with two major differences: 1) the total number of threads is ceiling(T, N) (l.8), and 2) each thread in the loop iterates over cells within its own chunk (l.19). Therefore, we have a different loop condition and different quantified invariants. Alpinist also supports this.

Above, each thread in the original template is assigned to one cell. The approach we describe here can easily be generalized to a kernel that accesses K cells. The resulting optimized kernel then accesses T $\times$ K kernels. A similar procedure can be applied as long as the tasks do not overlap, i.e., each cell is assigned to at most one thread.

### Kernel fusion

Kernel fusion is a GPU program optimization where we merge two or more consecutive kernels into one. It increases the potential to use thread-local registers to store intermediate results and can lead to less power consumption. See [3, 19, 26, 70, 71, 77] for the impact of kernel fusion on GPU programs.

### Template

**Fig. 24:**
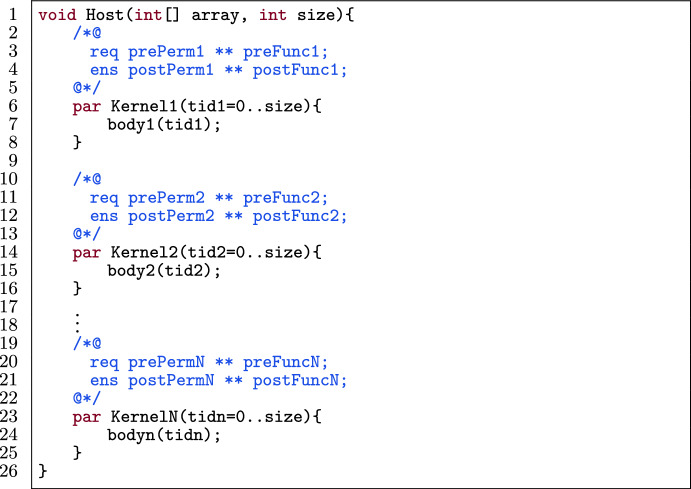
A kernel template for kernel fusion.

Figure 24 shows a template of N kernels in a verified GPU program. Each kernel consists of a body (l.7, 15, 24) and its contract (l.3–4, 11–12, 20–21). The contract is split up into pre- and postconditions and those are split up into permission-related conditions (prePerm and postPerm respectively) and functional-correctness conditions (preFunc and postFunc respectively).

A few assumptions are made regarding the kernels:

- The kernels are lock-free.
- The kernels do not lose permissions. A *permission loss* happens when a kernel requires permission p over a location, e.g., Perm(a[tid], 1), but does not ensure the same permission p for that location, e.g., Perm(a[tid], 1 $\backslash$ 2), in which case some permission is *lost*. However, the program is allowed to redistribute the permissions using a barrier, meaning each thread may get different permissions after the barrier, but the total permissions across all threads stay the same.
- A write permission is only specified if the kernel actually needs it.
- The array indices only use constants and thread identifier variables, using standard arithmetic operators (e.g., 2 $\times$ $tid$ + 1).

The first assumption restricts PVL to resemble GPU kernels more closely. The last three assumptions are to restrict the input of the algorithm discussed below.

### Transformation

The exact procedure by which the kernels are fused is heavily dependent on the preconditions and postconditions. It is difficult to give one template that covers all different possibilities. Thus, we separate the procedure of kernel fusion in three parts: 1) the permission-related preconditions, 2) permission-related postconditions and 3) the functional-correctness pre- and postconditions.

**Fig. 25:**
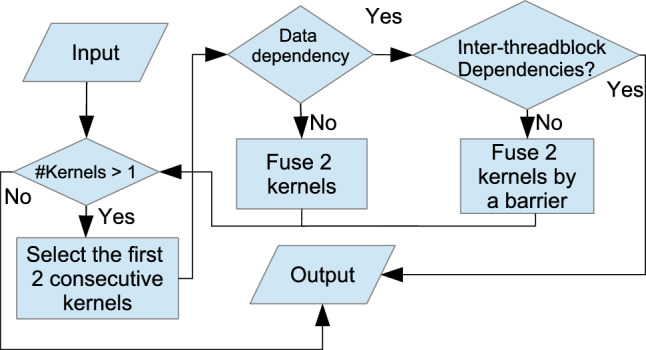
The general high-level procedure of kernel fusion optimization.

Figure 25 illustrates a high-level procedure of fusing kernels. The figure provides a generalized procedure to fuse an *arbitrary number* of consecutive kernels while considering *data dependency* and *thread-block dependencies* between them.

The thread-block data dependencies can be split into two categories, namely inter- and intra-thread-block data dependency. *Inter-thread-block data dependency* is data dependency between threads of different thread blocks and *intra-thread-block data dependency* is data dependency between threads within the same thread block.

The idea is to reduce consecutive kernels to a single kernel. Reduction in this context is to fuse the first two kernels and then repeatedly fuse the next kernel into the result of the previous fusion, eventually resulting in one kernel. In the $\texttt {n}^{th}$ iteration of the reduction, if there is no data dependency between the $\texttt {0..n}^{th}$ kernel^10^ and the $\texttt {n+1}^{th}$ kernel, we safely fuse them. Else if there is no inter-thread-block dependency, then we fuse the two kernels by inserting a barrier between the bodies. Else, there is inter-thread-block dependency, meaning kernel fusion is not possible and we should keep the remaining kernels unfused. That means the procedure terminates in two situations: 1) all kernels are fused, or 2) there is inter-thread-block data dependency between the $\texttt {0..n}^{th}$ kernel and $\texttt {n+1}^{th}$ kernel. In that case, the first $\texttt {n}$ kernels have been fused, and the remaining kernels will not be fused.

A benefit of this approach is that it only considers two kernels at a time. In this way, it can be determined whether a barrier is necessary between two specific kernels, and we do not miss any possible fusion optimization. Another benefit of this approach is that if inter-thread-block data dependency between two kernels 0..n and n+1 is detected, the output of the approach is the fusion of the $\texttt {0..n}^{th}$ kernels, and the remaining unfused kernels. This allows the user to not only identify an inter-thread-block data dependency between $\texttt {0..n}^{th}$ kernel and $\texttt {n+1}^{th}$ kernel, but also to obtain fused kernels where possible. The user can then decide whether to continue to fuse the remaining kernels (starting from the $\texttt {n+1}^{th}$ kernel).

There are multiple challenges in this transformation: (1) how to detect inter-thread-block data dependency between two kernels? (2) how to collect the pre- and postconditions for the fused kernel? and (3) how to combine permissions so that in the fused kernel the sum of the permissions for a location never exceeds 1?

**Fig. 26:**
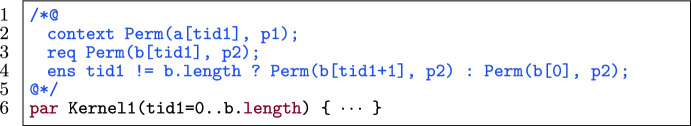
Simple kernel example.

**Fig. 27:**
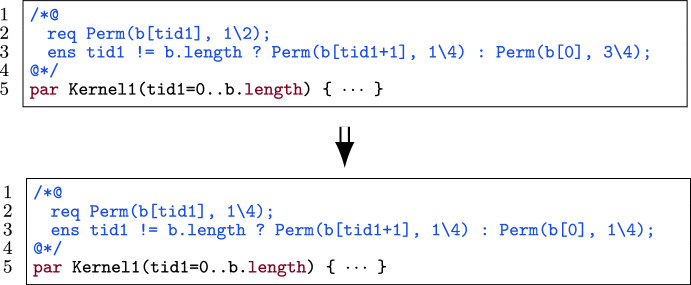
Example of changing the permissions in a kernel to have the same permissions.

The main difficulty in addressing these challenges is that we have to consider many different possible scenarios. Fortunately, we can use the information from the contracts of the two kernels. The permission patterns in the contracts (prePermN and postPermN, respectively) indicate for each thread which locations it reads from and writes to. If the applicability check phase succeeds, we continue with the procedure.

We provide the procedures to collect pre- and postconditions related to permissions and functional correctness in three parts, see Sects. 5.6.1, 5.6.2 and 5.6.3 respectively. Next, we provide a procedure to fuse the bodies of the two kernels possibly including an (annotated) barrier in Sect. 5.6.4. Moreover, we provide implementation details about the inter-thread-block data dependency detection in Sect. 5.6.5.

#### Functions over permission-annotation sets

Before explaining the different parts of the algorithm, we first introduce the concept of *permission-annotation sets* to aid reasoning about the permissions in a kernel contract. As the name suggests, a *permission-annotation set* is a set of permission-related annotation expressions. As an example, the permission-annotation set of the preconditions of the kernel in Fig. 26 is {Perm(a[tid1], p1), Perm(b[tid1], p2)} and the permission-annotation set for the postcondition is {Perm(a[tid1], p1),tid1 != b.length ? Perm(b[tid1+1], p2): Perm(b[0], p2)}.

For simplicity, we assume that permission annotations that are part of the same pattern have the same permissions. This is also a prerequisite of some of the functions defined below. In the example above, we see that the two permission annotations related to the shared variable b have the same permission. We argue that this assumption is not too restrictive. Any example where the permissions are not the same, can be altered such that the permissions are the same since any non-zero permission is a read permission. Figure 27 shows an example of a kernel with different read permissions (i.e. $1\backslash 4$, $1\backslash 2$ and $3\backslash 4$). By changing all the permission to, e.g., $1\backslash 4$, the permissions can be made the same across the same pattern. Moreover, in practice this assumption is generally true for examples verified using VerCors (including the experiments of the evaluation in Sect. 6).

We define five functions on these sets:

- $filter$(pas,vars) $=$ $\lbrace$ p $\mid$ p $\in$ pas $\wedge$ $contains$(p, vars) $\rbrace$
  Filters the annotations in the permission-annotation set pas that use at least one variable in vars. The $contains$(p,vars) function checks for the syntactical occurrence of the variables in vars in pas. E.g., 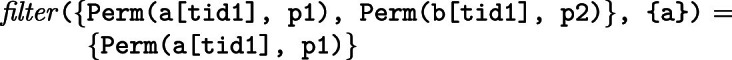
- $cperm$(pas): Returns the (concrete) permission from the permission-annotation set pas. This function requires that all permissions in the set are equal. E.g., 
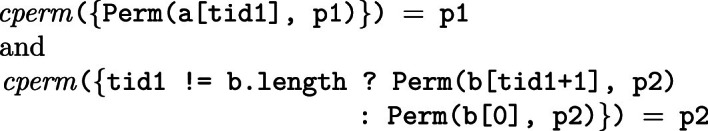
- $replaceAllPerm$(pas, newPerm) $=$ $\lbrace$ $replacePerm$(p, newPerm) $\mid$ p $\in$ pas $\rbrace$
  Replaces the permissions in the permission-annotation set pas with newPerm. The function $replacePerm$(p, newPerm) is a syntactical substitution of the permissions. E.g., 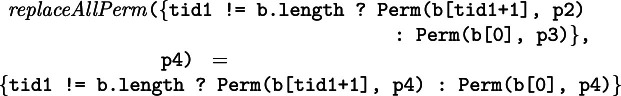
- $patt$(pas): Returns a set with the access patterns captured by the permission-annotation set pas. E.g., 
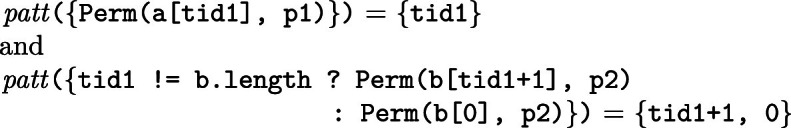
- $perm$(pas): Returns a mapping from array locations captured by the permission-annotation set pas to the amount of permissions captured in pas. This function requires that the permission-annotation sets only contain one array, which can be achieved using the $filter$ function, and that all permissions in the set are equal.
  E.g., suppose we have threads from 0 to b.length and the permission-annotation set 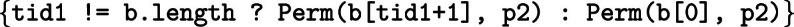 where only array b is used and all permissions are p2.
  For each location in array b, we can determine the permissions for that specific location, e.g., ,  (assuming b.length is at least 2), etc.
  Note the difference between a pattern and a location. A pattern, e.g., tid+1, points to a (concrete) location for a given tid. Different patterns can point to the same location for different tids, e.g., b[tid+1] for tid=0 is b[1] and b[tid] for tid=1 is also b[1]. This is important to consider when comparing kernels.
  Also note that the $perm$ function is not total. The cases relevant for the kernel fusion algorithm are covered, the other cases remain undefined. This also implies that all functions that use $perm$ in their definition are thereby also not total.
- $$ keys $$(pas)$$=$$ $$\lbrace $$ l $$\mid $$ (l,p) $$\in $$ $$ perm $$(pas) $$\rbrace $$
  Returns a set of locations (i.e., integer indices) that have permissions specified in pas (obtained using the $perm$ function).

We define three operators over the permission-annotation sets. Firstly, we define the union of two permission-annotation sets. Given two permission-annotation sets pas1 and pas2, pas1 $\cup$ pas2 is equivalent to the union operator on sets. In relation to the $perm$ function, the union operator behaves as follows:


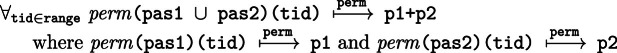


The thread identifier tid is taken from range, the range of possible thread identifiers (inferred from the kernel). In words, pas1 $\cup$ pas2 is defined as the sum of the permissions for each location. For example, suppose we have


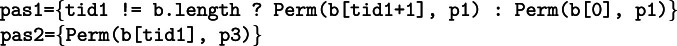


The set pas1 maps location b[1] (with tid=0) to permission p1 and pas2 maps location b[1] to permission p3, thus pas1 $\cup$ pas2 maps location b[1] to permission p1+p3.

Secondly $\le$, <, > and $=$ relations between permission-annotation sets are defined. Given two permission-annotation sets pas1 and pas2, we define


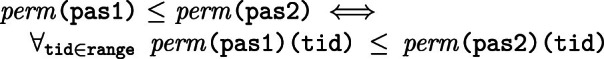


where range is the range of possible thread identifiers tid (inferred from the kernel). This can be read as; the permission for each location in pas1 is less than or equal to the permission for that location in pas2. The other operators are defined similarly. The syntax here is chosen to hide the complexity of the universal quantifier (over all locations), favouring readability of the algorithms.

Similarly, we define $\le$, <, > and $=$ relations between permission-annotation sets and a permission. Given a permission-annotation set pas and a permission p1, we define 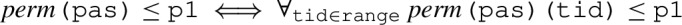


where range is the range of possible thread identifiers tid (inferred from the kernel). This can be read as; the permission that each location in pas points to is less than or equal to p1. The other operators are defined similarly.

Lastly, we define ${\mathop {\perp }\limits ^{sv}}$, disjointness between permission-annotation sets. 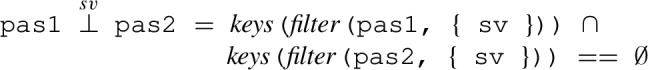


Two permission-annotation sets pas1 and pas2 are disjoint for a variable sv if the locations for the permissions for sv (i.e., the keysets of a mapping) in pas1 and pas2 are disjoint.

**Algorithm 1:**
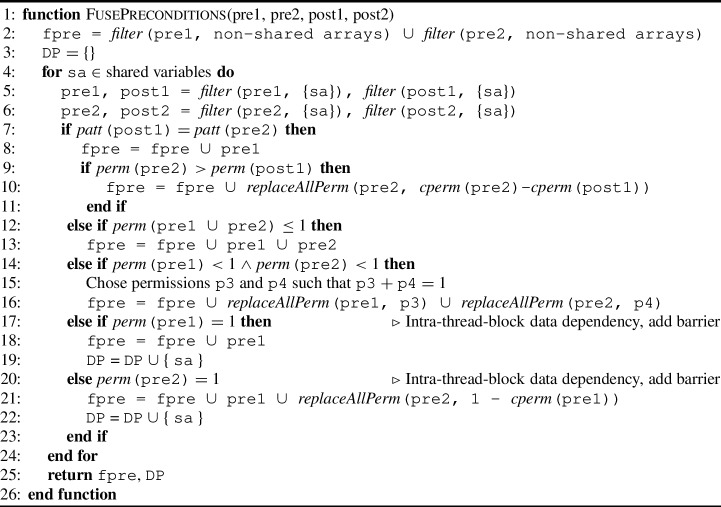
Kernel fusion procedure for collecting precondition permissions.

#### Permission-related preconditions

Algorithm 1 shows the essential steps to collect the preconditions related to permissions for array accesses of the fused kernel. It requires the pre- and postconditions (as permission-annotation sets) for the two kernels to fuse, namely pre1, post1, pre2, and post2 and returns a set with the preconditions of the fused kernel fpre and a set with data dependent shared variables DP to be used in Algorithm 3 and 4.

The algorithm works as follows. Each shared variable is considered individually (l.4). The postcondition of the first kernel post1 is compared to the precondition of the second kernel pre2 to understand how to add permissions of the preconditions of both kernels to the fused kernel. The reasoning behind initially comparing post1 to pre2, is to check whether post1 can (possibly) satisfy pre2 as is. If this is the case, we can take pre1 as the fused precondition since post1 follows from pre1.

If the patterns of post2 and pre1 are (syntactically) equivalent (l.7), then they refer to the same array cell. In this case, we add pre1 to the precondition of the fused kernel fpre (l.8). Then we check whether the permission in post1 is sufficient for pre2 (l.9). If this is not the case, we add the missing permission as well (l.10).

The remaining cases (l.12,14,17,20) in the algorithm correspond to the different edge cases that we should consider when the patterns of post1 and pre2 are not equivalent. In particular, there is intra-thread-block data dependency when the accumulated permission for one location is greater than 1, at least one kernel has write permission and there are no inter-thread-block dependencies. Therefore, we distinguish multiple cases: 1) the accumulated permission (in both kernels) for one location does not exceed 1 (l.12), 2) the accumulated permission exceeds 1, but no write permission is involved (l.14), or 3) and 4) at least one write is involved (l.17, 20).

In case 1, we collect the preconditions in both kernels (l.13). In case 2, we collect the precondition of both kernels, but we update the amount of permissions in such a way that the permissions for each array location is exactly 1 (l.15-16). In case 3, we only collect the precondition of $k1$, add sa to the set of data dependent shared variables DP and we add a barrier to redistribute the permissions (l.18). Finally in case 4, we add pre1, as well as pre2 with updated permission 1-$perm$(pre1), add sa to the set of data dependent shared variables DP and add a barrier (l.21, 22).

**Algorithm 2:**
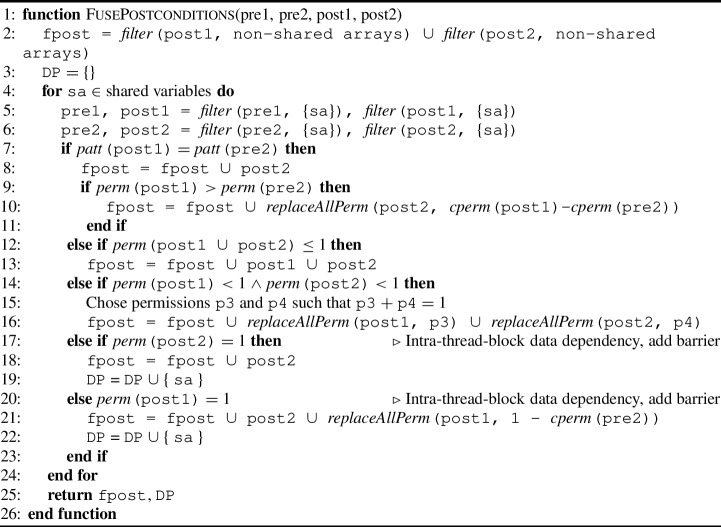
Kernel fusion procedure for collecting postcondition permissions.

##### Example

**Fig. 28:**
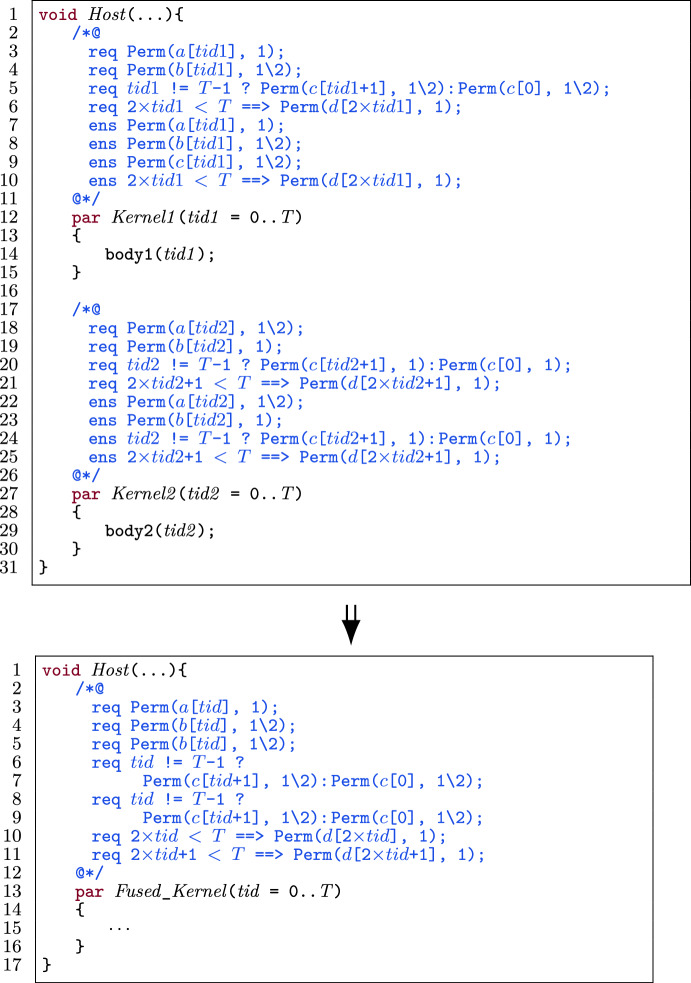
Example: Collecting permission preconditions in different cases when fusing two kernels.

Figure 28 shows an example of fusing the preconditions of two kernels. For the purpose of this example, we only show pre- and postcondition specifications related to permissions. There are four shared variables, namely $a$, $b$, $c$ and $d$. To collect permission preconditions in the fused kernel according to Algorithm 1, we follow the steps in lines 7-8 for variable $a$, the steps in lines 7-10 for variable $b$ and the steps in lines 12-13 for variable $d$. For variable $c$, we follow the steps in lines 20-22 and conclude that there is intra-thread-block data dependency. $\square$

#### Permission-related postconditions

Algorithm 2 shows the essential steps to collect the postcondition related to permissions for array accesses of the fused kernel. It again requires the pre- and postconditions (as permission-annotation sets) for the two kernels to fuse, namely pre1, post1, pre2, and post2 and returns a set with the postconditions of the fused kernel fpost and a set with data dependent shared variables DP to be used in Algorithm 3 and 4.

It is in spirit the same as Algorithm 1, but there are some differences: 1) lines 7-10 in Algorithm 2 are the dual of lines 7-10 in Algorithm 1, 2) in case of an intra-thread-block data dependency, if $perm$(pre2) is a write permission (l.17), we collect post2 (l.18). This is because we know that kernels do not lose permissions, i.e. $perm$(pre2) $=$ $perm$(post2), thus $perm$(post2) is a write permission. In case $perm$(pre2) is not a write permission but $perm$(pre1) is a write permission (l.20), we collect post2 (l.21). Moreover, we add post1 with the remaining permission 1-$perm$(pre2) (l.21). This comes from the fact that if the precondition of the fused kernel has write permission and there is no permission loss, thus fpost should return write permissions for each array location.

Note here that the check for $perm$(post2) being a write permission is before the check whether $perm$(post1) is a write permission. The check for post2 is first, because if $perm$(post2) is a write permission, it is added as the postcondition of the fused kernel, thereby ensuring all the acquired permissions from the precondition (Algorithm 1, l.21). Only if $perm$(post2) is a read permission and $perm$(post1) is a write permission, the extra permission is needed to be a write permission in total.

##### Example

**Fig. 29:**
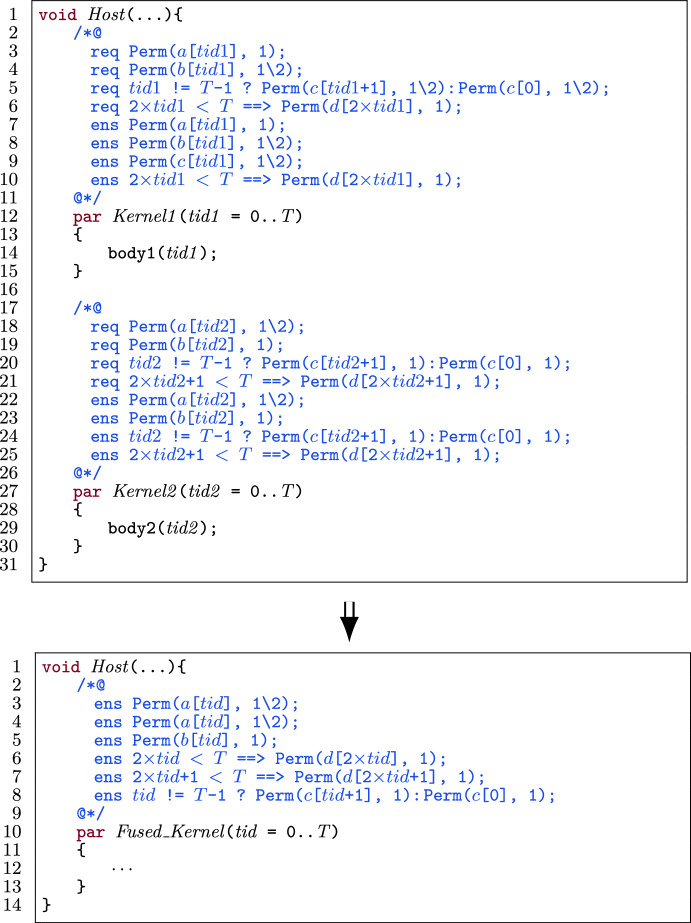
Example: Collecting permission postconditions in different cases when fusing two kernels.

Figure 29 shows the same example of fusing two kernels, but now focusing on permission postconditions. We follow Algorithm 2 to collect permission postconditions in the fused kernel for the four shared variables. For variable $a$, we go through the steps in lines 7-10, for variable $b$ the steps in lines 7-8 and for variable $d$ the step in lines 12-13. For variable $c$, we follow the steps in lines 17-19 in Algorithm 2. $\square$

#### Functional-correctness pre- and postconditions

Algorithm 3 shows how to collect the pre- and postconditions related to functional correctness. It requires the functional-correctness related pre- and postconditions (as permission-annotation sets) for the two kernels to fuse, namely pre1, post1, pre2, and post2, and a set of variables that have data dependencies DP, which is the union of the DP sets collected in Algorithms 1 and 2. Algorithm 3 returns the pre- and postconditions of the fused kernel ffpre and ffpost.

The algorithm first collects the preconditions (l.2-8) followed by the dual for the postconditions (l.10-17). Initially, the precondition of the first kernel pre1 is added to ffpre, as well as all annotations in pre2 related to non-shared arrays (l.2). These annotation are then removed from pre2 (l.3) to analyze further. Next, we investigate whether any (remaining) precondition in pre2 (where at least one shared array is specified) can be added to ffpre (l.4-8). For each precondition pre in pre2, we check whether 1) there are any arrays in pre that have intra-thread-block data dependency (using DP) and if so, whether 2) that variable has either been written to or whether 3) pre1 and pre2 are disjoint (l.5). This way we check whether pre has not been affected by the other kernel, and if so we can add it to ffpre. The steps for ffpost are similar in spirit (l.10-17) with the biggest difference that post2 is only added to ffpost afterwards (l.17) since post2 has to be checked after post1.

**Algorithm 3:**
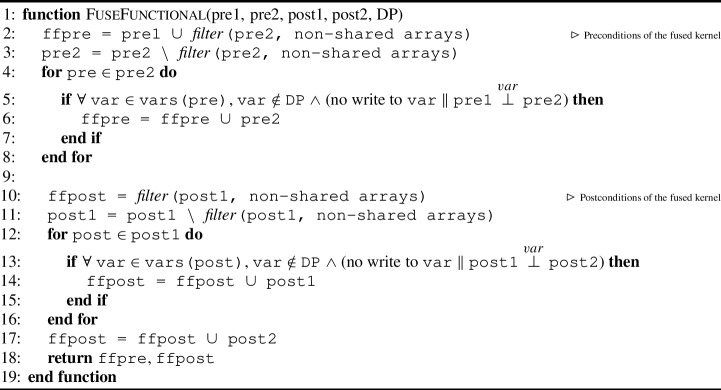
Kernel fusion procedure for collecting pre- and postcondition functional correctness.

##### Example

**Fig. 30:**
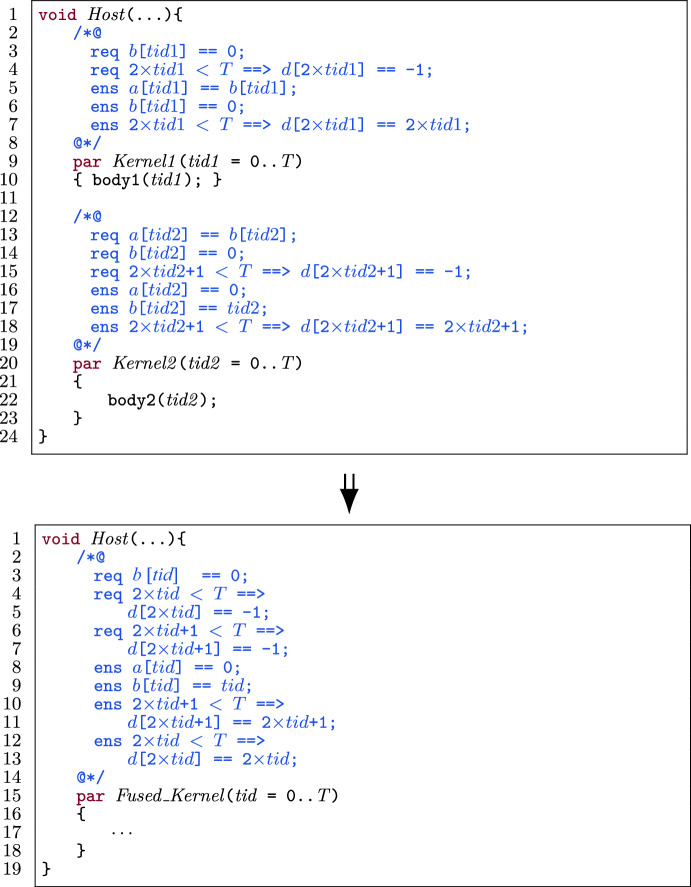
Collecting pre- and postcondition contract related to functional correctness in different cases when fusing two kernels.

Figure 30 shows an example of how to collect the contract for functional correctness. In this example we only represent specifications for functional correctness of arrays $a$, $b$ and $d$. We follow Algorithm 3 to add the contract for the three shared variables in the fused kernel. We add all preconditions of the first kernel (l.2) and all postconditions of the second kernel into the fused kernel (l.17). This corresponds to lines 3–5 and lines 8–11 in the fused kernel in Fig. 30. Then, for the rest of the contract, we follow the remaining steps of Algorithm 3. Concretely, for each precondition (related to functional correctness) in the second kernel, we check the condition (l.5) to add it to the fused kernel. In this example, we can only add 2$\times$ $tid2$+1 < *T* $\Rightarrow$ *d*[2$\times$ $tid2$+1] == -1, because according to the condition, there is no data dependency for *d* and both kernels access disjoint locations in *d*. For the two other preconditions in the second kernel, the condition does not hold, hence we do not collect them. In the same way, for the postconditions in the first kernel, we only add 2$\times$ $tid2$ < *T* $\Rightarrow$ *d*[2$\times$ $tid2$] == 2$\times$ $tid2$ (l.13) to the fused kernel and we do not collect the other two postconditions. $\square$

#### Fusing the bodies of the kernels

**Algorithm 4:**
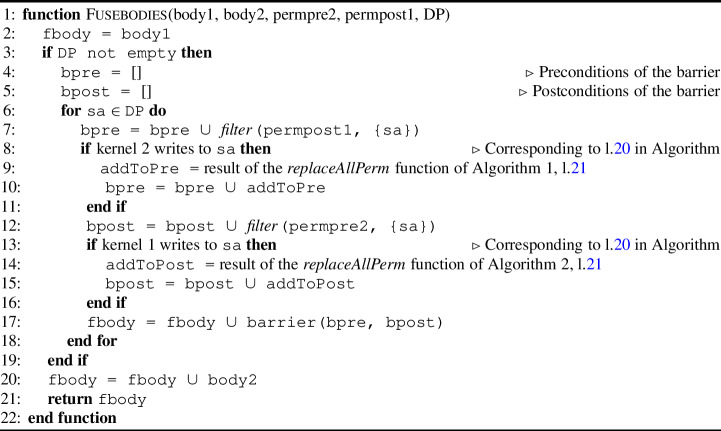
Kernel fusion procedure for fusing the bodies of the two kernels.

Finally, Algorithm 4 shows how to fuse the bodies of the two kernels. It requires the bodies of the two kernels body1 and body2 (which are both a list of statements), the permission-related precondition of the second kernel permpre2, the permission-related postcondition of the first kernel permpost1 and a set of variables that have data dependencies DP, as collected in Algorithm 1 and . Algorithm 4 returns the body of the fused kernel fbody.

fbody is initially set to body1 (l.2). If there is data dependency (l.3), a barrier is constructed that redistributes the permissions from the first kernel into the second kernel. Concretely, the precondition of this barrier bpre consists of all permission-related postconditions of kernel 1 permpost1 using the shared variable sa from DP (l.7). Similarly, the postcondition of this barrier bpost consists of all permission-related preconditions of kernel 2 permpre2 using the shared variable sa from DP (l.12). In addition, the result of the $replaceAllPerm$ functions from Algorithm 1 and 2 are added as pre- and postconditions of the barrier (l.10 and 15).

To explain the reason why the result of these algorithms is added as a pre/postcondition of the barrier, we first focus on l.20 of Algorithm 1. In this case, kernel 2 has write permission and consequently kernel 1 does not have write permission. To satisfy this write permission (for the fused kernel), we add the precondition of kernel 1 (i.e., a read permission, l.21) and add the remaining permission (i.e., the result of the $replaceAllPerm$) such that the sum of the two is 1, i.e., a write permission. The same reasoning also holds for Algorithm 1, l.20.

This reasoning is also applied in Algorithm 4. For bpre we first take the postcondition of kernel 1 (l.7), which is a read permission and for bpost we take the precondition of kernel 1 (l.12), which is a write permission. Since bpre is a read permission and bpost is a write permission, we need additional permission in bpre to satisfy bpost. This missing permission corresponds to the result of the $replaceAllPerm$ in Algorithm 1, l.22.

Finally, body2 is appended to fbody (l.20) and fbody is returned (l.21).

**Fig. 31:**
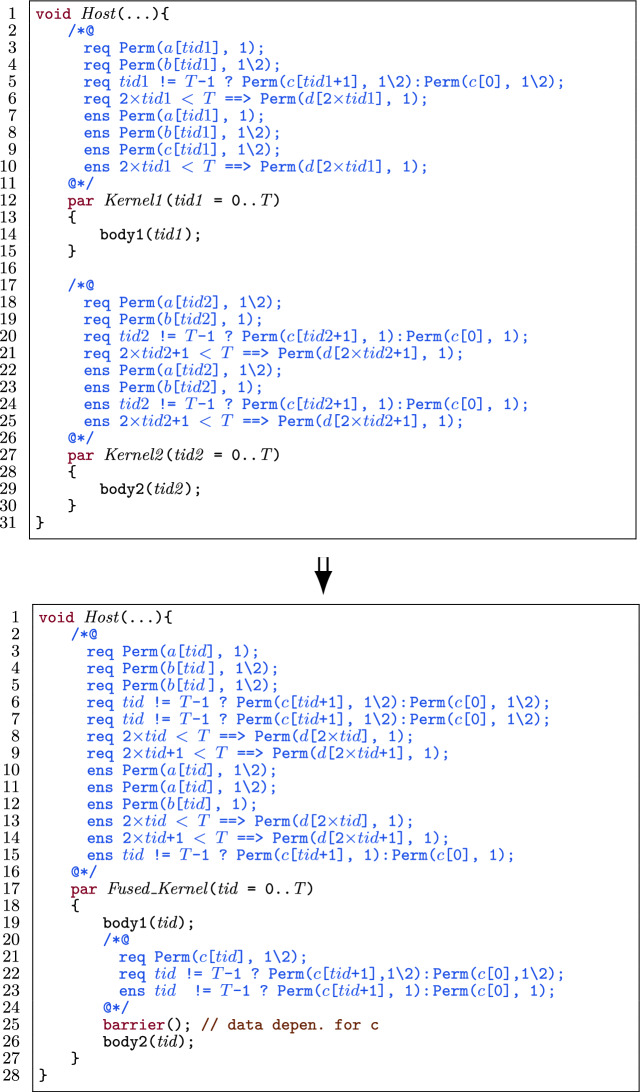
Fusing two kernels using an annotated barrier.

##### Example

Figure 31 shows the same example of fusing two kernels using an (annotated) barrier. We also represent the contract related to permissions to explain how to collect permission specification in the barrier. As there are intra-thread-block data dependencies, we can fuse the two kernels by inserting a barrier between the bodies (Fig. 31, l.25). We follow the steps in l.2-12 and l.20 in Algorithm 4. Concretely, in the contract of the barrier, we add whatever postcondition for $c$ is in the first kernel as the precondition in the barrier (l.21). The body of the first kernel requires the precondition on l.6 in the fused kernel and redistributes it to l.21 in the contract of the barrier (corresponding to the postcondition of the first kernel on l.9). The other precondition in the barrier (l.22) comes from the other precondition for $c$ in the fused kernel (l.7). Moreover, we add the precondition for $c$ in the second kernel as the postcondition in the barrier (l.23). In this way, the first body has the required permissions and the barrier redistributes the permissions to be used by the second body. $\square$

#### Implementing inter-thread-block data dependency detection

One of the implementation challenges of kernel fusion is to check inter-thread-block data dependency in the applicability checking phase. The check consists of two parts: 1) A check for data dependency and 2) a check for thread-block dependencies. With these two checks, we can determine whether we have data dependencies between two kernels and if so, whether this data dependency is an inter-thread-block or intra-thread-block dependency.

Our idea of detecting kernel dependencies is similar to detecting loop iteration dependencies, see [1]. To detect data dependency for a specific shared array, the function SV is used. Given an array a and a kernel specification, SV outputs a mapping from array indices to the permission specified for that index in the kernel specification. This function is in essence the implementation of the $perm$ function over permission-annotation sets, where $perm$ is defined more abstractly.

**Fig. 32:**
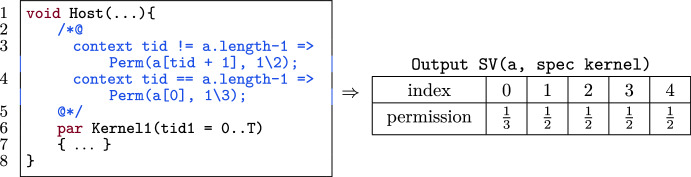
Example output of the SV function for array a.

Figure 32 shows an example of the output of SV. The kernel has 1$\backslash$2 permission for a[tid+1] and 1$\backslash$3 permission for a[0] if tid+1 is out of bounds. SV takes an array name and the pre- and postconditions of a kernel (of the form $\texttt {cond(tid) => Perm(a[patt(tid)], p)}$) on l.3 and l.4, and returns a mapping from indices patt(tid) to the permissions p (in Fig. 32: right).

If the function SV is executed for two kernels to fuse with the same shared array a, the results SV$_1$(a) and SV$_2$(a) can be compared to determine whether there is data dependency between the two kernels. This comparison is described generally at l.12-l.22 in Algorithm 1. For each corresponding location in SV$_1$(a) and SV$_2$(a), we can determine, for example, whether both permissions combined do not exceed 1 (l.12) or whether the location in k1 has write permission (l.17).

Once we know there is data dependency, we can check whether it is an inter-thread-block dependency using the SV function. The size of the thread blocks TS is given as input from the user. Using SV, we determine for a given shared variable which range of indices has been accessed by both kernels. If this range is larger than TS (i.e., the thread block size), then the fused kernel has inter-thread-block dependencies.

## Evaluation

This section describes the evaluation of this paper’s approach using Alpinist. The goal is to

**Q1**: test whether the approach works on GPU programs.
**Q2**: investigate how long it takes for Alpinist to transform GPU programs and how this affects the verification time.
**Q3**: investigate the usability of the approach on real-world examples.

### Setup of the experiments

Alpinist is applied on examples from three different sources. The first source consists of hand-made examples that cover different scenarios for each optimization. The second source is a collection of verified programs from VerCors’ example repository.^11^ The third source consists of complex case studies that are already verified in VerCors: two parallel prefix sum algorithms [55], parallel stream compaction and summed-area table algorithms [53], a variety of sorting algorithms [54], a solution [28] to the VerifyThis 2019 challenge 1 [18] and a Tic-Tac-Toe example [64] based on [23].

In total, we applied the optimizations 30 times in the first category, 23 times in the second category and 17 times in the third category (in total 70 experiments). All the examples are annotated with special optimization annotations such that Alpinist can apply those optimizations automatically. Some examples from the second and third source required additional annotations in order to apply Alpinist ’s optimizations, namely eight examples for iteration merging and five examples for tiling. For these examples, the total number of thread was specified as a pre-/postcondition of the form "nT ==..." where nT is a variable representing the total number of threads. All other examples could be optimized by Alpinist without changes to the example. All these examples are publicly available at [59]. All experiments were conducted on a MacBook Pro 2020 (macOS 11.3.1) with a 2.0GHz Intel Core i5 CPU. Each experiment was performed ten times, after which the average times, i.e., optimization and verification times, of those executions were recorded for the experiment.

### Summary of the results & discussion

**Q1** To test whether the approach works on GPU programs, we applied one of the six optimizations in all 70 experiments and used VerCors to reverify all the resulting programs. All these tests were successful.

To test whether the applicability checking phase works as intended, 49 out of 70 mentioned experiments have been altered such that the experiments verify with VerCors, but fail to optimize with Alpinist. The alterations were of two kinds: Firstly, we removed annotations required by the optimization, but not necessarily the verification of the program. These alterations have been mentioned in the previous section as "required additional annotations to apply Alpinist ’s optimizations". Secondly, the parameters of the special optimization annotations have been changed to not pass the applicability checking phase or one of the requirements of the algorithm.

**Fig. 33:**
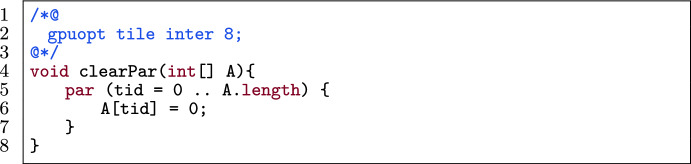
A verifying example that is not possible to tile with Alpinist due to a missing annotation.

**Fig. 34:**
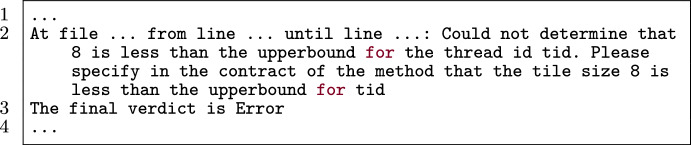
Output of Alpinist when applying tiling to Fig. 33^12^.

Concretely, we give an example for each kind of alteration. Figure 33 is a kernel to be tiled. Line 2 specifies that Alpinist is going to apply inter-tiling with tile size 8. To apply this optimization, the total number of threads needs to be known, which in this case is equivalent to A.length. The original file does not specify anything about A.length. Since A.length is not a concrete constant, Alpinist cannot apply the optimization and returns an error message as seen in Fig. 34.

**Fig. 35:**
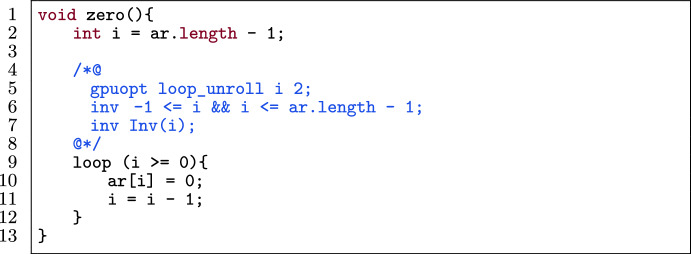
A verifying example that is not possible to unroll with Alpinist.

**Fig. 36:**
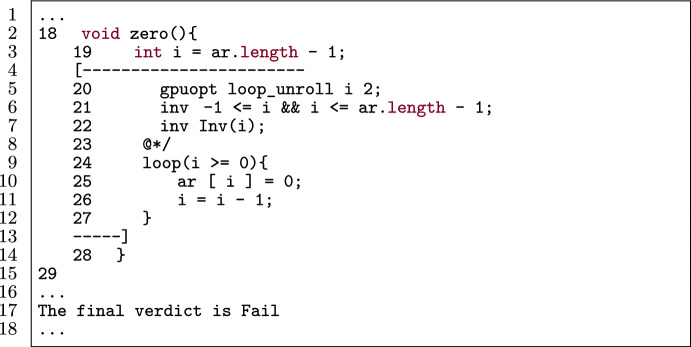
Output of Alpinist when applying loop unrolling to Fig. 35. (Altered to match code/line numbers of the simplified PVL example in Fig. 35).

Figure 35 is a kernel with a loop to be unrolled. Line 5 specifies that Alpinist is going to apply loop unrolling with iteration variable i and unroll factor 2. Without an annotation regarding the length of a, which determines the initial value of the iteration variable i, Alpinist is not able to determine that the loop is unrollable. This results in the error message as seen in Fig. 36, pointing to the loop that could not be unrolled.

**Q2** To investigate how long it takes for Alpinist to transform GPU programs, we recorded the transformation time for each optimization applied to all the examples.

**Table 1:** A summary of the optimization and verification times for all optimizations

		Loop unrolling	Iteration merging	Data prefetching	Matrix linearization	Tiling	Kernel fusion
Optimization time (s)	Min.	0.048	0.027	0.012	0.045	0.053	0.118
	Max.	0.172	0.217	0.027	0.095	0.062	0.360
	Avg.	0.091	0.093	0.019	0.057	0.056	0.193
	Med.	0.075	0.081	0.017	0.054	0.055	0.153
Verification time orig. (s)	Min.	1.050	6.750	10.514	9.625	10.674	13.302
	Max.	53.907	48.695	16.058	19.664	13.373	66.991
	Avg.	17.557	16.112	13.846	12.544	11.858	21.466
	Med.	13.936	12.780	13.521	12.402	11.419	14.816
Verification time opt. (s)	Min.	7.389	7.128	10.585	9.769	13.373	12.366
	Max.	48.870	55.368	16.110	19.921	19.578	14.896
	Avg.	18.906	17.999	13.613	12.612	15.898	13.709
	Med.	14.477	13.314	13.125	12.508	14.155	13.969
Difference between orig & opt.	Min. (%)	604	6	1	1	25	−7
	Max. (%)	−9	14	0	1	46	−78
	Avg. (%)	8	12	−2	1	34	−36
	Med. (%)	4	4	−3	1	24	−6

**Fig. 37:**
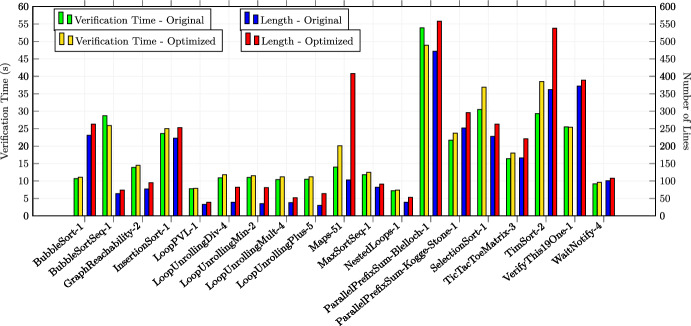
The evaluation on the loop unrolling optimization with the number of unrollings as a suffix.

**Fig. 38:**
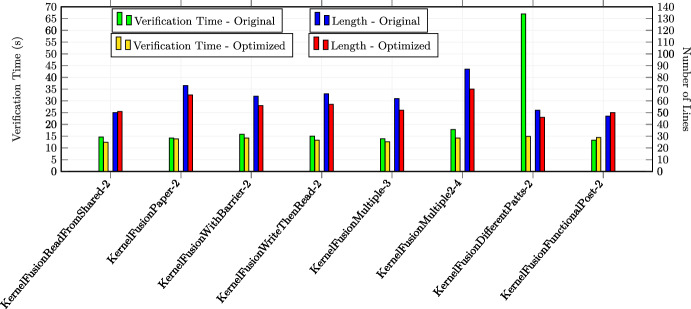
The evaluation on the kernel fusion optimization.

**Fig. 39:**
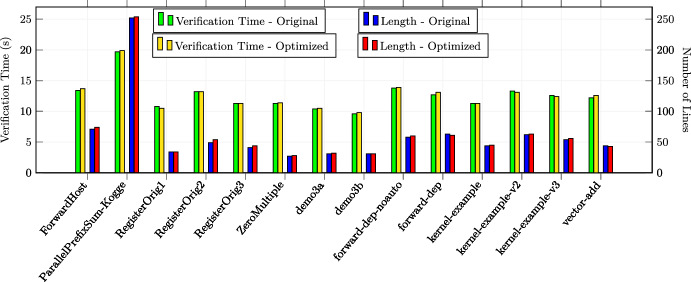
The evaluation on the data prefetching optimization.

**Fig. 40:**
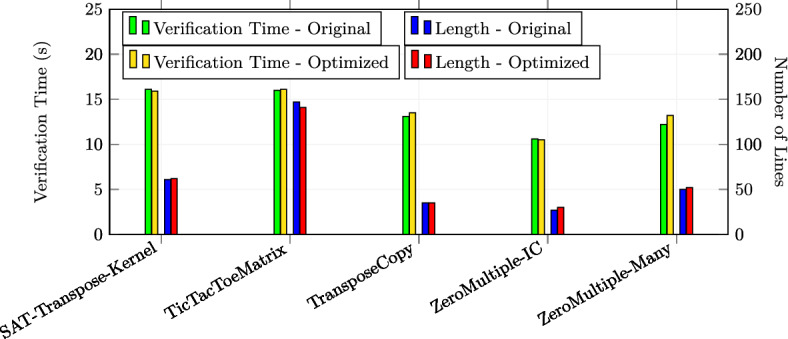
The evaluation on the matrix linearization optimization.

**Fig. 41:**
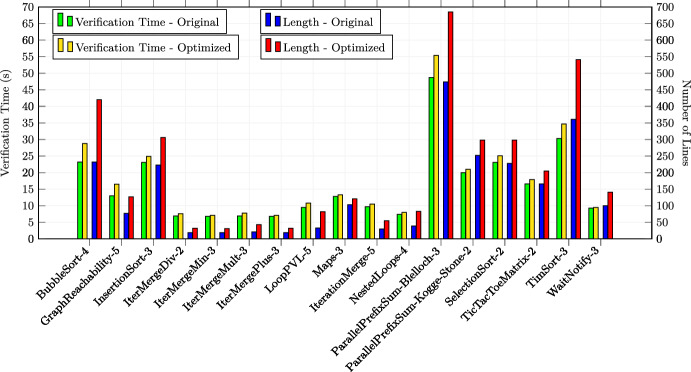
The evaluation on the iteration merging optimization with the number of mergings as a suffix.

**Fig. 42:**
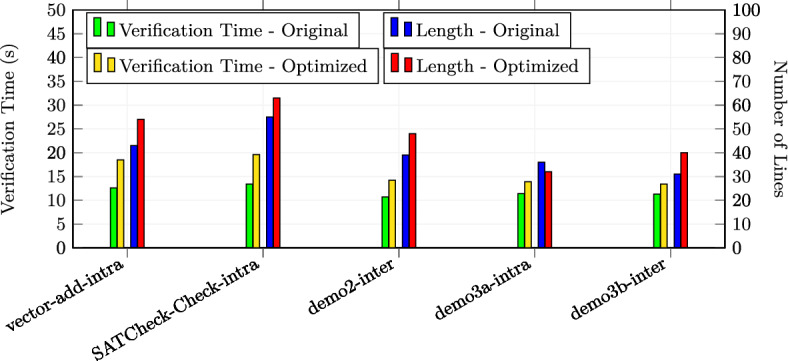
The evaluation on the tiling optimization.

Table 1 summarizes the best and worst optimization times for the six optimizations (as reported by Alpinist). To investigate the impact on the verification time, the table also shows the (best and worst) verification times of the original and optimized programs (as reported by VerCors). For comparison, the difference (in percentage) between the original and optimized is also shown. The table shows the minimum, maximum, average and median times of all examples. The results for the individual cases can be found in Figs. 37, 38, 39, 40, 41 and 42. The examples with results that deviate from the trends mentioned above are discussed in Sect. 6.3.

It can be observed that Alpinist takes insignificant time to apply each optimization to all the examples. The verification time after optimizing generally increases. For loop unrolling, tiling and iteration merging, the verification time increases. This can be attributed to the additional code that is generated. For matrix linearization and data prefetching, the verification time slightly increases. This can be attributed to the linear expressions in matrix linearization and the extra statements to read from/write to the registers in data prefetching.

For kernel fusion, the trend is a slight decrease in verification time, however, the last two experiments (as seen in Fig. 38) differ from this trend. Sections 6.3.1 and 6.3.2 analyze these two experiments and investigate how why they differ from the other six experiments. With the conclusions of those experiments, we see that kernel fusion generally decreases the verification time slightly. Other factors, such as those explained in Sects. 6.3.1 and 6.3.2, can have an effect on the verification time.

**Q3** To investigate the usability of the approach on real-world examples, we successfully applied it on the third category with the complex case studies. Table 2 shows the average optimization and verification times of applying loop unrolling, iteration merging, matrix linearization and data prefetching to these case studies. Note that in the case studies only these four optimizations were applicable, kernel fusion and tiling were not applicable. In the table, N/A indicates that the optimization is not applicable to the example.

**Table 2:** An overview of optimizing case studies, where **#** is the unroll factor (for loop unrolling) or the merge factor (for iteration merging), **OT** the time it takes to optimize, **VB** the original verification time (Verification Before) and **VA** the optimized verification time (Verification After). All times are in seconds

Case	Loop unrolling				Iter. merging				Matrix lin.			Data pref.		
	#	OT	VB	VA	#	OT	VB	VA	OT	VB	VA	OT	VB	VA
BubbleSort [54]	1	0.090	28.3	27.0	4	0.154	24.5	30.1	N/A	N/A	N/A	N/A	N/A	N/A
InsertionSort [54]	1	0.105	24.5	25.2	3	0.183	22.9	25.2	N/A	N/A	N/A	N/A	N/A	N/A
SelectionSort [54]	1	0.095	25.8	26.9	2	0.207	25.1	77.6	N/A	N/A	N/A	N/A	N/A	N/A
TimSort [54]	2	0.144	29.2	34.4	3	0.123	46.4	32.7	N/A	N/A	N/A	N/A	N/A	N/A
Blelloch [55]	1	0.161	69.8	48.8	3	0.243	48.8	56.1	N/A	N/A	N/A	N/A	N/A	N/A
Kogge-Stone [55]	1	0.118	21.7	23.1	2	0.078	22.4	23.7	N/A	N/A	N/A	0.091	21.4	21.1
TicTacToe[64]	3	0.111	18.7	20.8	2	0.081	17.4	18.8	N/A	N/A	N/A	N/A	N/A	N/A
VerifyThis [28]	1	0.099	27.5	34.0	N/A	N/A	N/A	N/A	N/A	N/A	N/A	N/A	N/A	N/A
Transpose [53]	N/A	N/A	N/A	N/A	N/A	N/A	N/A	N/A	0.026	16.0	16.0	N/A	N/A	N/A

### Profiling of deviating examples

In Q2, it was observed that for each optimization there is a general trend in verification time. From the 70 experiments, there are three particular experiments that deviate from these trends, namely the experiment *KernelFusionDifferentPatts-2* and *KernelFusionFunctionalPost-2* for the kernel fusion optimization and the experiment *ParallelPrefixSum-Blelloch-1* for the loop unrolling optimization. In this section, we profile these examples and investigate how and why they differ from the trend.

The setup for the profiling is as follows: 1) The examples are run using VerCors version 1.4, 2) the resulting Viper file is saved, and 3) the profiling is performed on the generated Viper file with the most recent developer version of VerCors 2.0 [68]. The reason for using VerCors version 1.4 is to stay consistent with the evaluation above, since the VerCors transformations on the input file could influence the result. Thus, we use VerCors 1.4 to generate the resulting Viper file. This file is then given as input to VerCors 2.0 since the profiling functionality is implemented in this version.^12^

#### KernelFusionDifferentPatts-2

**Fig. 43:**
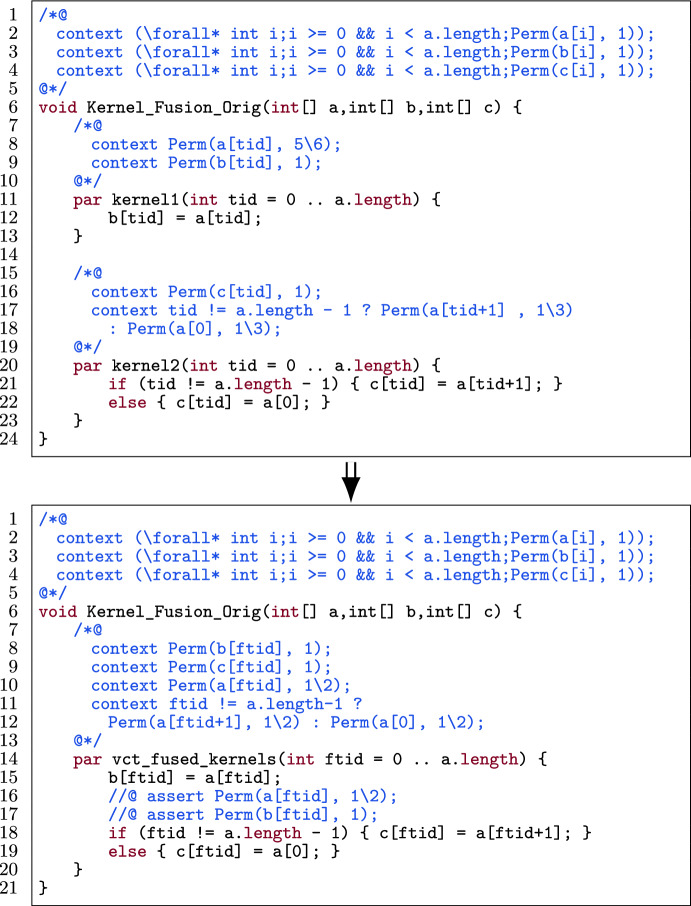
Application of kernel fusion on the KernelFusionDifferentPatts example.

Figure 43 shows the original example and the optimized file after applying kernel fusion. The functional-correctness related annotations have been omitted from Fig. 43 to focus on the differences.

The original program can be summarized as follows: The host function requires write permission for all items in arrays a, b and c. The first kernel specifies read permission to items in a and write permission to items in b after which the second kernel specifies read permission to items in a (with different access patterns) and write permission to items c. After this the host function ensures write permission to all items in a, b and c.

**Fig. 44:**
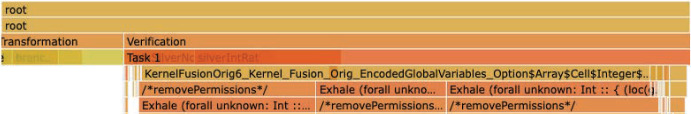
The flame chart of the difference between the original and optimized KernelFusionDifferentPatts example.

The original and optimized example were profiled and the flame chart in Fig. 44 was produced. This flame chart is the difference between the profiling data of the optimized and the original example, i.e., for each reported task (e.g., the Verification and (VerCors’) Transformation) the difference in time between the optimized and the original example.

From Fig. 44, we read that VerCors spends a significant amount of time on the verification of the postcondition of the host function (l. 2). This corresponds to the three Exhale statements in Fig. 44 under the Verification task. The profiling reports that the total difference in verification time is -36,4 s and that this postcondition takes up 33,0 s of it.

We know that this postcondition is the reason for different behavior with respect to the other experiments. However, the question remains why this postcondition is difficult to prove. To investigate this, we have tried different variants of the program and we made a comparison between this example and the other examples in the evaluation of kernel fusion.

The result of the investigation can be summarized as follows. In this specific example, VerCors does not seem to retain the fact that the three arrays a, b and c are distinct, i.e., the arrays are not aliases and the arrays are not overlapping. Therefore, VerCors takes a signification amount of time proving the postcondition, as is has to be reproven.

To retain this information over the two kernels, we add Perm(c[tid], 1) to the contract of kernel1 and Perm(b[tid], 1) to the contract of kernel2. With these annotations, VerCors can more easily prove that the three arrays are distinct. With these changes, the original verification time becomes 35,399 s and the optimized verification time 28,264 s.

The conclusion of this investigation is that this example does not behave differently from the other examples due to the kernel fusion optimization, but due to a lack of annotations that guide the prover. Such annotations are present in the other six examples, but not present in this specific example. By adding those annotations, the verification time is more in line with the other examples.

#### KernelFusionFunctionalPost-2

**Fig. 45:**
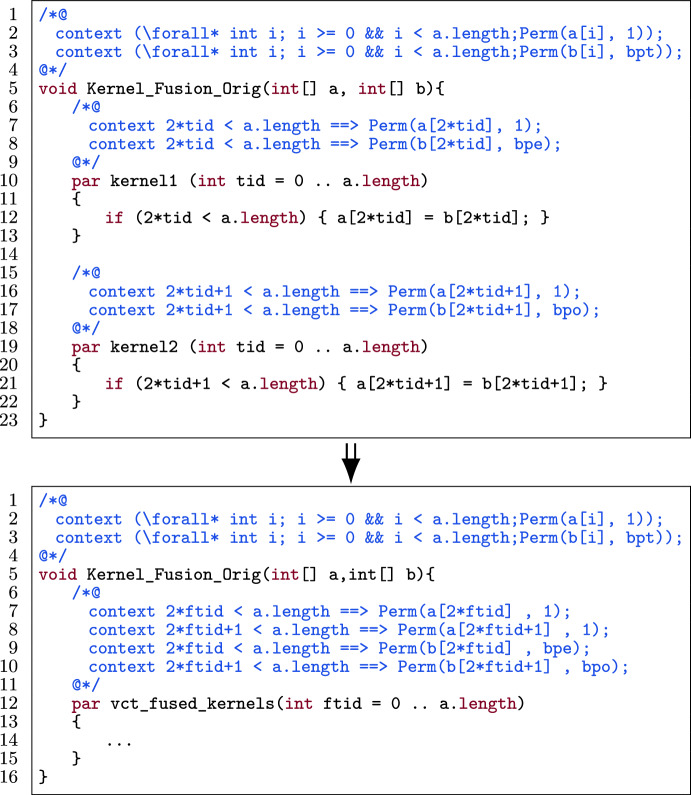
Application of kernel fusion on the KernelFusionFunctionalPost example.

Figure 45 shows the original example and the optimized file after applying kernel fusion. Annotations irrelevant to the explanation below have been omitted from Fig. 45 to focus on the question at hand.

The original program can be summarized as follows: The host function requires write permission for all items in array a and some permission bpt for all items in b. Both kernels specify write permission to the items in a, the first kernel specifies some permission bpe to the even locations of b and the second kernel specifies some permission bpo to the oneven locations of b. The bodies of the kernels are not relevant for the explanation.

There are three permissions left as variables in the unoptimized and optimized code, namely bpt (l. 3), bpe (l. 8) and bpo (l. 17) that correspond to the total permission for the locations in b, the permission specific to the even locations in b and the permission specific to the off locations. The experiment in Fig. 38 had bpt equal to 1, and bpe and bpo equal to $1\backslash 2$.

To understand what happens in this experiment, we tried different combinations of these variables. Table 3 shows nine different combinations of these three variables under the column *Setup 1*. The table reports the verification time of the original file and the verification time of the optimized file, which are averages of ten runs.

**Table 3:** Different setups for the KernelFusionFunctionalPost example and their verification times

		Setup 1		Setup 2	
		Verification time orig. (s)	Verification time opt. (s)	Verification time orig. (s)	Verification time opt. (s)
Case 1	**bpt** $=1$	31,862	34,117	32,642	31,307
	**bpe** $=1$				
	**bpo** $=1$				
Case 2	**bpt** $=1$	31,524	33,956	32,220	30,329
	**bpe** $=1\backslash 2$				
	**bpo** $=1\backslash 2$				
Case 3	**bpt** $=1$	30,681	33,403	32,471	30,606
	**bpe** $=1\backslash 4$				
	**bpo** $=1\backslash 4$				
Case 4	**bpt** $=1$	31,465	33,684	32,411	31,804
	**bpe** $=3\backslash 4$				
	**bpo** $=3\backslash 4$				
Case 5	**bpt** $=1\backslash 2$	31,289	32,744	32,425	31,481
	**bpe** $=1\backslash 2$				
	**bpo** $=1\backslash 2$				
Case 6	**bpt** $=1\backslash 2$	30,702	33,440	33,212	32,182
	**bpe** $=1\backslash 4$				
	**bpo** $=1\backslash 4$				
Case 7	**bpt** $=3\backslash 4$	31,111	32,990	33,998	31,935
	**bpe** $=3\backslash 4$				
	**bpo** $=3\backslash 4$				
Case 8	**bpt** $=3\backslash 4$	30,814	32,697	33,100	31,903
	**bpe** $=1\backslash 2$				
	**bpo** $=1\backslash 2$				
Case 9	**bpt** $=3\backslash 4$	31,716	32,875	32,597	31,487
	**bpe** $=1\backslash 4$				
	**bpo** $=1\backslash 4$				

From *Setup 1*, we notice that the permissions of b do not have an effect on the verification time. From inspection of the flame charts of the different combinations, we notice that the original program spends time in proving that the arrays are distinct, similar to what we have seen in Sect. 6.3.1. In particular, this experiment is different, since there are two patterns related to b do not overlap, namely 2$\times$tid and 2$\times$tid+1. VerCors seems to spend some time in proving that the permission for each location is not more than 1 across the kernel invocations and the postcondition.

Thus, we altered the program with the suggested changes in the conclusion of Sect. 6.3.1, where additional permissions were added to retain specific information. Given the original program in Fig. 45, we added the pre- and postconditions of kernel one on lines 7 and 8 to kernel two (l.19) and the pre- and postconditions of kernel two on lines 16 and 17 to kernel one (l.10).

The experiments were ran again with all the different combinations of *Setup 1* and are reported in Table 3 under the column *Setup 2*. We notice for these experiments that the verification time decreases across all combinations.

The result of this investigation can be summarized as follows. The increase in verification time has a similar explanation to the conclusion of Sect. 6.3.1 where VerCors spends time in proving that the arrays are not aliases and that the different patterns of b do not result in more than one permission. By adding the above-mentioned annotations, we give the prover more information to easily prove the non-aliasing.

The conclusion of this investigation is the same as the conclusion of Sect. 6.3.1. This example does not behave differently from the other examples due to the kernel fusion optimization itself, but due to a lack of annotations that guide the prover. Such annotations are present in the other six examples, but not present in this specific example. By adding those annotations, the verification time is more in line with the other examples.

#### ParallelPrefixSum-Blelloch-1

This example is an implementation of Blelloch’s parallel prefix sum algorithm [55]. Due to the length of the files, we cannot show the entire algorithm itself.

The result for ParallelPrefixSum-Blelloch-1 as we see in Fig. 37 decreases in verification time as opposed to the other loop unroll examples that increase in verification time. Besides being opposed to the trend of the other examples, this result also conflicts with the same experiment in [52], where the verification time increases. In other words, the example seems to be unstable across different setups.

To investigate this, we profiled this example in two different setups; 1) the setup for profiling as described above and 2) the setup for profiling using only VerCors 2.0.

Table 4 shows the difference between the optimized and original verification time for both setups. The profiling functionality shows the total run time of VerCors in 4 phases: Parsing, Name Resolution, VerCors’ Transformation and Verification. The first three phases are performed within VerCors and the last phase is performed by Viper and Z3. Here, Verification (with a capital V) refers to the time spent by Viper and Total to the total run time of VerCors (as reported in Fig. 37). The table can be read as follows: Parsing for setup 1 is 16,38 s slower, i.e., the optimized example was 16,38 s slower then the original example for setup 1.

From the Total time, we read that setup 1 increases in total run time and setup 2 decreases in total run time. For both setups, there is an increase in VerCors’ Transformation time and a decrease in Verification. The increase in VerCors’ Transformation time can be attributed to the generated code for loop unrolling, which is to be expected. The decrease in Verification time is unexpected, since we expected that the increase in (generated) code and annotations would also increase the verification time.

**Table 4:** Total verification time for ParallelPrefixSum-Blelloch-1 for different setups

	Setup 1 (s) VerCors 1.4 & 2.0	Setup 2 (s) VerCors 2.0
Parsing	16,38	−1,6
Name Resolution	0,061	0,073
VerCors’ Transformation	64,46	22,54
Verification	−8,48	−25,63
Total Run Time	72,61	−5,52

**Fig. 46:**
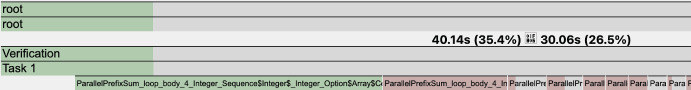
The flame chart of setup 1 for the ParallelPrefixSum-Blelloch example.

**Fig. 47:**
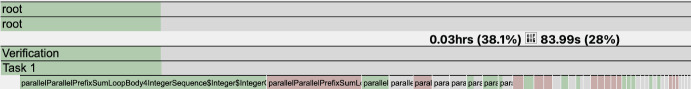
The flame chart of setup 2 for the ParallelPrefixSum-Blelloch example.

Figures 46 and 47 show the flame charts for setup 1 and 2 respectively,^13^ specifically focused on the Verification phase. The charts are read as follows: the top row named root is the entire verification, the third row named Verification (and the fourth row Task 1) is the same Verification time as in Table 4 and the row at the bottom are the individual verification tasks (in this case prefixed with *ParallelPrefixSum...* and *parallelParallelPrefixSum...* respectively). The green rows represent time saved and the red rows represent time lost. The subgoals are cut away for readability.

From the flame charts, we read that both setups saved time on some verification subgoals (i.e. the green rows) and lost time for other verification subgoals (i.e. the red rows), but overall the result is a net time save, seen from the Verification row. From this investigation, we conclude that the results for the two setups follow different trends mainly due to VerCors’ transformation phase. Depending on the transformation phase, the trend that we observe is either an increase or decrease in total run time.

To completely understand where this difference comes from, we would need to profile VerCors’ transformations and investigate how the individual transformations perform and come up with a solution to reduce the effect of VerCors’ transformations. This is planned for future work.

## Related work

To the best of our knowledge, this is the first paper to showcase a tool that implements annotation-aware transformations. We categorize the related work into three parts, covering both tools and optimizations.

### Automatic optimizations without correctness

There is a large body of related work, see e.g., [3, 5, 16, 19, 27, 30, 38, 51, 70, 71, 77–79], that shows the impact of automated optimizations on GPU programs, but does not consider *correctness*, or the preservation of it. Our tool can potentially complement these approaches by preserving the provability of the optimized programs.

### Correctness proofs for transformations

Another body of related work focuses on different approaches to preserve provability not specific to GPU programs. CompCert [33, 34] is a formally verified C compiler that preserves semantic equivalence of the source and compiled program, by proving correctness of each transformation in the compilation process. Wijs and Engelen [75] and De Putter and Wijs [49] prove the preservation of functional properties over transformations on models of concurrent systems. They prove preservation of model-independent properties. This approach differs from ours as they work on models instead of concrete programs.

### Compiler optimization correctness

Finally, there is related work that focuses on the compilation of sequential programs, performing transformations from high-level source code to lower-level machine code while preserving the semantics. These approaches either consider no parallelization, or are specific to one architecture. In GPU programming, the optimizations often need to be applied manually rather than during the compilation process.

Namjoshi and Xu [45] use a proof checker to show equivalence between an original WebAssembly program and an optimized program. An equivalence proof is generated based on the transformations. Namjoshi and Singhania [44] created a semi-automatic loop optimizer with user-directives. The loops are verified during compilation. For each transformation, semantics are defined to guarantee semantical equivalence to the original program. Namjoshi and Pavlinovic [43] focus on recovering from precision loss due to semantics-preserving program transformations and propose systematic approaches to simplify analysis of the transformed program. Finally Gjomemo et al. [20] help compiler optimizations by supplying high-level information gathered by external static analysis (e.g., Frama-C). This information is used by the compiler for better reasoning.

## Conclusion

In this paper, we presented an approach to automatically apply optimizations to GPU programs while preserving provability by defining annotation-aware transformations. Given an unoptimized, annotated GPU program, we showed how our approach transforms both the code and the annotations, with the goal to preserve the provability of the optimized GPU program.

The approach has been implemented in the Alpinist tool. Alpinist supports loop unrolling, iteration merging, matrix linearization, data prefetching, tiling and kernel fusion. We discussed the design and implementation of Alpinist, and we validated it by verifying a set of examples and reverifying their optimized counterparts.

### Future work

There are other optimizations that could be supported, such as data prefetching for all memory patterns as mentioned by [5] and the optimizations mentioned by [26]. There is also the question whether these optimizations could be applied without user-specified annotations. Another open question is if and how this approach can be used in program compilation. We also plan to extend this approach to preserve the provability of transpiled code, e.g., CUDA to OpenCL conversions. Moreover, we plan to investigate how Alpinist can be combined with techniques such as *autotuning* that automatically detect the potential for applying specific optimizations and identify optimal parameter configurations [4, 72].

Another possible continuation of this work is to investigate how the approach in this paper can be used in combination with a stepwise refinement approach as presented by [31].

## Footnotes

1 We refer to Section 5 of [26] for a detailed explanation of the GPU architecture and the GPU programming abstractions.

2 In practice, the size of a block cannot exceed a specific upper-bound, but for this example, we assume that a.length is sufficiently small.

3 Since the development of Alpinist, using VerCors 1.4, some of these aspects such as multiple thread blocks, floating point numbers and shared memory have been implemented in VerCors 2.0.

4 If c was negative, for the multiplication and division, i would oscillate between positive and negative values and hence would not always be useful as an array index. Hence we consider c to be positive.

5 This check is necessary since the unrolled loop bodies are not guarded by if statements.

6 Iteration merging is also referred to as loop unrolling/vectorization in the literature.

7 To focus on the main ideas of iteration merging, we omit some of the assertions in Fig. 8 that are not relevant for this optimization.

8 Ghost variables are not part of the code and are used for verification purposes.

9 Since N is in the range 1 $\le$ N $\le$ T, the last chunk might have fewer cells.

10 We use $\texttt {0..n}^{th}$ kernel to denote the result of fusing the $\texttt {0}^{th}$ upto $\texttt {n}^{th}$ kernel.

11 The example repository of VerCors is available at https://github.com/utwente-fmt/vercors/tree/dev/examples.

12 An explanation of the profiling functionality of VerCors 2.0 can be found here https://github.com/utwente-fmt/vercors/wiki/Profiling

13 Unfortunately, the flame charts produced by the profiling tool (similar to Fig. 44) are not readable. Thus we show an alternative representation of the flame charts, also provided by the profiling tool.

## References

[1] Allen R, Kennedy K (1987) Automatic translation of Fortran programs to vector form. ACM Trans Program Lang Syst (TOPLAS) 9(4):491–542

[2] Amighi A (2018) Specification and verification of synchronisation classes in java: A practical approach. PhD thesis, University of Twente

[3] Ashari A, Tatikonda S, Boehm M et al (2015) On optimizing machine learning workloads via kernel fusion. ACM SIGPLAN Notices 50(8):173–182

[4] Ashouri A, Killian W, Cavazos J et al (2018) A survey on compiler autotuning using machine learning. ACM Computing Surveys 51(5): 96:1–96:42

[5] Ayers G, Litz H, Kozyrakis C, et al (2020) Classifying memory access patterns for prefetching. In: Proceedings of the Twenty-Fifth International Conference on Architectural Support for Programming Languages and Operating Systems, pp 513–526

[6] Bardsley E, Donaldson A (2014) Warps and atomics: Beyond barrier synchronization in the verification of GPU kernels. In: NASA Formal Methods, LNCS, vol 8430. Springer, pp 230–245

[7] Bell N, Garland M (2008) Efficient sparse matrix-vector multiplication on CUDA. Tech. rep., Citeseer

[8] Berdine J, Calcagno C, O’Hearn P (2005) Smallfoot: Modular Automatic Assertion Checking with Separation Logic. In: de Boer F, Bonsangue M, Graf S, et al (eds) FMCO, LNCS, vol 4111. Springer, Berlin, Heidelberg, pp 115–137

[9] Bertolli C, Betts A, Mudalige G, et al (2011) Design and performance of the OP2 library for unstructured mesh applications. In: Proceedings of the 1st Workshop on Grids, Clouds and P2P Programming (CGWS), Lecture Notes in Computer Science, vol 7155. Springer, Berlin, Heidelberg, pp 191–200, 10.1007/978-3-642-29737-3_22

[10] Betts A, Chong N, Donaldson A et al (2012) GPUVerify: a verifier for GPU kernels. OOPSLA. ACM, New York, USA, pp 113–132

[11] Blom S, Huisman M, Mihelčić M (2014) Specification and verification of GPGPU programs. Sci Comput Program 95:376–388

[12] Bornat R, Calcagno C, O’Hearn P, et al (2005) Permission accounting in separation logic. In: Proceedings of the 32nd ACM SIGPLAN-SIGACT symposium on Principles of programming languages (POPL), pp 259–270

[13] Boyland J (2003) Checking Interference with Fractional Permissions. SAS, LNCS, vol 2694. Springer, Berlin, Heidelberg, pp 55–72

[14] Catanzaro B, Keller A, Garland M (2014) A decomposition for in-place matrix transposition. ACM SIGPLAN Notices 49(8):193–206

[15] Collingbourne P, Cadar C, Kelly PH (2011) Symbolic testing of OpenCL code. In: Haifa Verification Conference, Springer, pp 203–218

[16] Cowan M, Maleki S, Musuvathi M, et al (2022) Gc3: An optimizing compiler for GPU collective communication. arXiv preprint, https://www.microsoft.com/en-us/research/publication/gc3-an-optimizingcompiler-for-gpu-collective-communication/

[17] DeFrancisco R, Cho S, Ferdman M et al (2020) Swarm model checking on the GPU. International Journal on Software Tools for Technology Transfer 22:583–599. 10.1007/s10009-020-00576-x

[18] Dross C, Furia CA, Huisman M, et al (2021) Verifythis 2019: a program verification competition. International Journal on Software Tools for Technology Transfer pp 1–11

[19] Filipovič J, Madzin M, Fousek J et al (2015) Optimizing CUDA code by kernel fusion: application on BLAS. The Journal of Supercomputing 71(10):3934–3957

[20] Gjomemo R, Namjoshi KS, Phung PH, et al (2015) From verification to optimizations. In: International Workshop on Verification, Model Checking, and Abstract Interpretation, Springer, pp 300–317

[21] Grauer-Gray S, Xu L, Searles R, et al (2012) Auto-tuning a High-Level Language Targeted to GPU Codes. In: Proc. 2012 Innovative Parallel Computing (InPar), IEEE, pp 1–10, 10.1109/InPar.2012.6339595

[22] van den Haak LB, Wijs A, van den Brand M, et al (2020) Formal methods for GPGPU programming: is the demand met? In: Proceedings of the 16th International Conference on Integrated Formal Methods (IFM 2020), Lecture Notes in Computer Science, vol 12546. Springer, Berlin, Heidelberg, pp 160–177, 10.1007/978-3-030-63461-2_9

[23] Hamers R, Jongmans SS (2020) Safe sessions of channel actions in Clojure: a tour of the discourje project. In: International Symposium on Leveraging Applications of Formal Methods, Springer, pp 489–508

[24] He M, Vafeiadis V, Qin S, et al (2016) Reasoning about fences and relaxed atomics. In: 2016 24th Euromicro International Conference on Parallel, Distributed, and Network-Based Processing (PDP), IEEE, pp 520–527

[25] Herrmann F, Silberholz J, Tiglio M (2011) Black Hole Simulations with CUDA. In: GPU Computing Gems Emerald Edition. Morgan Kaufmann, Burlington, Massachusetts, chap 8, p 103–111

[26] Hijma P, Heldens S, Sclocco A, et al (2023) Optimization techniques for GPU programming. ACM Computing Surveys 55(11). 10.1145/3570638

[27] Hong C, Sukumaran-Rajam A, Nisa I, et al (2019) Adaptive sparse tiling for sparse matrix multiplication. In: Proceedings of the 24th Symposium on Principles and Practice of Parallel Programming, pp 300–314

[28] Huisman M, Joosten S (2019) A solution to VerifyThis 2019 challenge 1. https://github.com/utwente-fmt/vercors/blob/97c49d6dc1097ded47a5ed53143695ace6904865/examples/verifythis/2019/challenge1.pvl

[29] Huisman M, Blom S, Darabi S, et al (2018) Program correctness by transformation. In: 8th International Symposium On Leveraging Applications of Formal Methods, Verification and Validation (ISoLA), LNCS, vol 11244. Springer, Berlin, Heidelberg

[30] Konstantinidis A, Kelly PH, Ramanujam J, et al (2013) Parametric GPU code generation for affine loop programs. In: International Workshop on Languages and Compilers for Parallel Computing, Springer, pp 136–151

[31] Lammich P (2022) Refinement of parallel algorithms down to LLVM. In: 13th International Conference on Interactive Theorem Proving (ITP 2022), Schloss Dagstuhl-Leibniz-Zentrum für Informatik

[32] Le Q, Ngiam J, Coates A, et al (2011) On Optimization Methods for Deep Learning. In: Proceedings of the 28th International Conference on Machine Learning (ICML). Omnipress, New York, USA, pp 265–272

[33] Leroy X (2006) Formal certification of a compiler back-end or: programming a compiler with a proof assistant. In: Conference record of the 33rd ACM SIGPLAN-SIGACT Symposium on Principles of Programming Languages, pp 42–54

[34] Leroy X (2009) A formally verified compiler back-end. Journal of Automated Reasoning 43(4):363–446

[35] Li G, Gopalakrishnan G (2010) Scalable SMT-based verification of GPU kernel functions. SIGSOFT FSE 2010, Santa Fe, NM. USA. ACM, New York, USA, pp 187–196

[36] Li G, Li P, Sawaya G, et al (2012) GKLEE: concolic verification and test generation for GPUs. In: ACM SIGPLAN Notices, ACM, pp 215–224

[37] Lindholm L, Nickolls J, Oberman S et al (2008) NVIDIA Tesla: a unified graphics and computing architecture. IEEE Micro 28(2):39–55. 10.1109/MM.2008.31

[38] Liou JY, Wang X, Forrest S, et al (2020) Gevo: GPU code optimization using evolutionary computation. ACM Transactions on Architecture and Code Optimization 17(4). 10.1145/3418055

[39] Liu X, Tan S, Wang H (2012) Parallel statistical analysis of analog circuits by GPU-accelerated graph-based approach. In: Proceedings of the 2012 Conference and Exhibition on Design, Automation & Test in Europe (DATE). IEEE Computer Society, Washington, DC, pp 852–857, 10.1109/DATE.2012.6176615

[40] de Moura LM, Bjørner N (2008) Z3: An efficient SMT solver. In: Ramakrishnan C, Rehof J (eds) TACAS, LNCS, vol 4963. Springer, Berlin, Heidelberg, pp 337–340

[41] Müller P, Schwerhoff M, Summers A (2016) Viper–a verification infrastructure for permission-based reasoning. In: VMCAI

[42] Murthy GS, Ravishankar M, Baskaran MM, et al (2010) Optimal loop unrolling for GPGPU programs. In: 2010 IEEE International Symposium on Parallel & Distributed Processing (IPDPS), IEEE, pp 1–11

[43] Namjoshi KS, Pavlinovic Z (2018) The impact of program transformations on static program analysis. In: International Static Analysis Symposium, Springer, pp 306–325

[44] Namjoshi KS, Singhania N (2016) Loopy: Programmable and formally verified loop transformations. In: International Static Analysis Symposium, Springer, pp 383–402

[45] Namjoshi KS, Xue A (2021) A self-certifying compilation framework for WebAssembly. In: International Conference on Verification, Model Checking, and Abstract Interpretation, Springer, pp 127–148

[46] OpenCL (2011) The OpenCL 1.2 specification

[47] Osama M, Wijs A (2019) Parallel SAT Simplification on GPU architectures. In: TACAS, Part I, LNCS, vol 11427. Springer, Berlin, Heidelberg, pp 21–40

[48] Osama M, Wijs A, Biere A (2021) SAT solving with GPU accelerated Inprocessing. In: Proceedings of the 27th International Conference on Tools and Algorithms for the Construction and Analysis of Systems (TACAS), Part I, Lecture Notes in Computer Science, vol 12651. Springer, Berlin, Heidelberg, pp 133–151, 10.1007/978-3-030-72016-2_8

[49] de Putter S, Wijs A (2016) Verifying a verifier: on the formal correctness of an LTS transformation verification technique. In: International Conference on Fundamental Approaches to Software Engineering, Springer, pp 383–400

[50] Ragan-Kelley J, Barnes C, Adams A et al (2013) Halide: a language and compiler for optimizing parallelism, locality, and recomputation in image processing pipelines. ACM Sigplan Notices 48(6):519–530

[51] Rocha RC, Pereira AD, Ramos L et al (2017) Toast: automatic tiling for iterative stencil computations on GPUs. Concurrency and Computation: Practice and Experience 29(8):e4053

[52] Safari M (2022) Correct optimized GPU programs. PhD Thesis–Research UT, graduation UT, University of Twente, Netherlands, 10.3990/1.9789036553421

[53] Safari M, Huisman M (2020a) Formal verification of parallel stream compaction and summed-area table algorithms. In: International Colloquium on Theoretical Aspects of Computing, Springer, pp 181–199

[54] Safari M, Huisman M (2020b) A generic approach to the verification of the permutation property of sequential and parallel swap-based sorting algorithms. In: International Conference on Integrated Formal Methods, Springer, pp 257–275

[55] Safari M, Oortwijn W, Joosten S, et al (2020) Formal verification of parallel prefix sum. In: NASA Formal Methods Symposium, Springer, pp 170–186

[56] Safari M, Oortwijn W, Huisman M (2021) Automated verification of the parallel Bellman-Ford algorithm. In: Drăgoi C, Mukherjee S, Namjoshi K (eds) Static Analysis. Springer International Publishing, Cham, pp 346–358

[57] Şakar Ö (2020) Extending support for axiomatic data types in VerCors. http://essay.utwente.nl/80892/

[58] Şakar Ö, Safari M, Huisman M, et al (2022a) Alpinist: An Annotation-Aware GPU Program Optimizer, Springer, Berlin, Heidelberg, p 332–352. Lecture Notes in Computer Science, 10.1007/978-3-030-99527-0_18

[59] Şakar Ö, Safari M, Huisman M, et al (2022b) The repository for the examples used in Alpinist. https://github.com/OmerSakar/Alpinist-Examples.git

[60] Shimobaba T, Ito T, Masuda N et al (2010) Fast calculation of computer-generated-hologram on AMD HD5000 series GPU and OpenCL. Optics Express 18(10):9955–9960 20588849 10.1364/OE.18.009955

[61] Sundfeld D, Havgaard JH, Gorodkin J, et al (2017) CUDA-Sankoff: using GPU to accelerate the pairwise structural RNA alignment. In: 2017 25th Euromicro International Conference on Parallel, Distributed and Network-based Processing (PDP), IEEE, pp 295–302

[62] The CUDA team (2021) Documentation of the CUDA unroll pragma. https://docs.nvidia.com/cuda/cuda-c-programming-guide/index.html#pragma-unroll, accessed Oct. 6, 2021

[63] The Halide team (2021) Documentation of the Halide unroll function. https://halide-lang.org/docs/class_halide_1_1_func.html#a05935caceb6efb8badd85f306dd33034, accessed Oct. 6, 2021

[64] TicTacToeMatrixGrid (2022) The verification of tictactoe program. https://github.com/utwente-fmt/vercors/blob/0a2fdc24419466c2d3b7a853a2908c37e7a8daa7/examples/session-generate/MatrixGrid.pvl

[65] Unkule S, Shaltz C, Qasem A (2012) Automatic restructuring of GPU kernels for exploiting inter-thread data locality. In: International Conference on Compiler Construction, Springer, pp 21–40

[66] Van Werkhoven B, Maassen J, Bal HE et al (2014) Optimizing convolution operations on GPUs using adaptive tiling. Futur Gener Comput Syst 30:14–26

[67] VerCors1.4 (2024) VerCors version 1.4. https://github.com/utwente-fmt/vercors/tree/v1

[68] VerCors2.0 (2023) The developer branch of VerCors version 2.0. https://github.com/utwentefmt/vercors/tree/0b6a3b2eb21192198647548bcb9cb9b559a1ed64

[69] Viper team (2016) Viper project website. http://www.pm.inf.ethz.ch/research/viper, http://www.pm.inf.ethz.ch/research/viper

[70] Wahib M, Maruyama N (2014) Scalable kernel fusion for memory-bound GPU applications. In: SC’14: Proceedings of the International Conference for High Performance Computing, Networking, Storage and Analysis, IEEE, pp 191–202

[71] Wang G, Lin Y, Yi W (2010) Kernel fusion: an effective method for better power efficiency on multithreaded GPU. In: 2010 IEEE/ACM Int’l Conference on Green Computing and Communications & Int’l Conference on Cyber, Physical and Social Computing, IEEE, pp 344–350

[72] Van Werkhoven B (2019) Kernel tuner: a search-optimizing GPU code auto-tuner. Futur Gener Comput Syst 90:347–358

[73] Wienke S, Springer P, Terboven C, et al (2012) OpenACC–first experiences with real-world applications. In: Proceedings of the 18th European Conference on Parallel and Distributed Computing (EuroPar), Lecture Notes in Computer Science, vol 7484. Springer, Berlin, Heidelberg, pp 859–870, 10.1007/978-3-642-32820-6_85

[74] Wijs A (2016) BFS-based model checking of linear-time properties with an application on GPUs. In: CAV, Part II, LNCS, vol 9780. Springer, Berlin, Heidelberg, pp 472–493

[75] Wijs A, Engelen L (2014) REFINER: towards formal verification of model transformations. In: NFM. Springer, Cham, LNCS, pp 258–263

[76] Wijs A, Neele T, Bošnački D (2016) GPUexplore 2.0: unleashing GPU explicit-state model checking. In: Proceedings of the 21st International Symposium on Formal Methods, Lecture Notes in Computer Science, vol 9995. Springer, Berlin, Heidelberg, pp 694–701, 10.1007/978-3-319-48989-6_42

[77] Wu H, Diamos G, Wang J, et al (2012) Optimizing data warehousing applications for GPUs using kernel fusion/fission. In: 2012 IEEE 26th International Parallel and Distributed Processing Symposium Workshops & PhD Forum, IEEE, pp 2433–2442

[78] Xu C, Kirk SR, Jenkins S (2009) Tiling for performance tuning on different models of GPUs. In: 2009 Second International Symposium on Information Science and Engineering, IEEE, pp 500–504

[79] Yang Y, Xiang P, Kong J et al (2010) A GPGPU compiler for memory optimization and parallelism management. ACM Sigplan Notices 45(6):86–97

